# Microbial biodiversity assessment of the European Space Agency’s ExoMars 2016 mission

**DOI:** 10.1186/s40168-017-0358-3

**Published:** 2017-10-25

**Authors:** Kaisa Koskinen, Petra Rettberg, Rüdiger Pukall, Anna Auerbach, Lisa Wink, Simon Barczyk, Alexandra Perras, Alexander Mahnert, Diana Margheritis, Gerhard Kminek, Christine Moissl-Eichinger

**Affiliations:** 1 Department for Internal Medicine, Section of Infectious Diseases and Tropical Medicine, Medical University of Graz, Graz, Austria; 2 BioTechMed-Graz, Graz, Austria; 3 Radiation Biology Department, German Aerospace Center (DLR), Institute of Aerospace Medicine, Cologne, Germany; 4 Leibniz-Institute DSMZ - German Collection of Microorganisms and Cell Cultures, Braunschweig, Germany; 5 Department for Microbiology, University of Regensburg, Regensburg, Germany; 6 Institute of Environmental Biotechnology, Graz University of Technology, Graz, Austria; 7 Thales Alenia Space, Turin, Italy; 8 European Space Agency, Noordwijk, The Netherlands

**Keywords:** ExoMars, Planetary protection, Life-detection, Astrobiology, Cleanroom microbiota

## Abstract

**Background:**

The ExoMars 2016 mission, consisting of the Trace Gas Orbiter and the Schiaparelli lander, was launched on March 14 2016 from Baikonur, Kazakhstan and reached its destination in October 2016. The Schiaparelli lander was subject to strict requirements for microbial cleanliness according to the obligatory planetary protection policy. To reach the required cleanliness, the ExoMars 2016 flight hardware was assembled in a newly built, biocontrolled cleanroom complex at Thales Alenia Space in Turin, Italy. In this study, we performed microbiological surveys of the cleanroom facilities and the spacecraft hardware before and during the assembly, integration and testing (AIT) activities.

**Methods:**

Besides the European Space Agency (ESA) standard bioburden assay, that served as a proxy for the microbiological contamination in general, we performed various alternative cultivation assays and utilised molecular techniques, including quantitative PCR and next generation sequencing, to assess the absolute and relative abundance and broadest diversity of microorganisms and their signatures in the cleanroom and on the spacecraft hardware.

**Results:**

Our results show that the bioburden, detected microbial contamination and microbial diversity decreased continuously after the cleanroom was decontaminated with more effective cleaning agents and during the ongoing AIT. The studied cleanrooms and change room were occupied by very distinct microbial communities: Overall, the change room harboured a higher number and diversity of microorganisms, including *Propionibacterium*, which was found to be significantly increased in the change room. In particular, the so called alternative cultivation assays proved important in detecting a broader cultivable diversity than covered by the standard bioburden assay and thus completed the picture on the cleanroom microbiota.

**Conclusion:**

During the whole project, the bioburden stayed at acceptable level and did not raise any concern for the ExoMars 2016 mission. The cleanroom complex at Thales Alenia Space in Turin is an excellent example of how efficient microbiological control is performed.

**Electronic supplementary material:**

The online version of this article (10.1186/s40168-017-0358-3) contains supplementary material, which is available to authorized users.

## Background

Finding life outside the terrestrial biosphere is one of the drivers for humans to venture beyond Earth. The reports on numerous habitable planets have fuelled the speculations and hopes for the existence of extra-terrestrial life ([1]; NASA Exoplanet Archive; http://exoplanetarchive.ipac.caltech.edu/). However, these potentially life-bearing planets are far beyond human’s reach—except Mars, where the special regions provide conditions in terms of temperature and water activity that would allow the propagation of terrestrial life on Mars today [2, 3].

No Martian life forms are yet known, but the observation of gaseous methane outbreaks in the Martian atmosphere has raised tremendous interest. Methane was frequently detected in the thin Martian atmosphere by the Planetary Fourier Spectrometer on ESA’s Mars Express, or other Earth- and Mars-based instruments [4, 5]. On Earth, methane is an excellent signature for microbial activity: More than 90% of the methane detected on Earth has been produced by microorganisms [6]. On Mars, the origin of methane is still subject of speculation. Potential sources are production in magma, the serpentinization of basalt (olivine, pyroxene)—or the activity of methanogenic microorganisms in a permafrost-like setting [6]. However, methane can remain archived in frozen reservoirs in form of methane clathrate for long periods of time, so if the origin is biogenic, the time point of potential microbial methane production remains unclear.

To date, numerous missions have been sent to Mars during the last decades to find life, signatures thereof or suitable environmental conditions for microbial life. NASA’s Viking program (1975), the Mars Exploration Rover Mission (2003), the Phoenix lander (2008), and the Mars Science Laboratory including Curiosity rover (2012) were still not able to return clear positive results. However, with the ability to explore extra-terrestrial environments directly with spacecraft there comes great responsibility: contamination of the instruments and potential extra-terrestrial ecosystem by accidentally transferred terrestrial microorganisms could tremendously affect the actual, but also future scientific missions and the planetary body itself.

In order to limit the microbial contamination via space missions, obligatory rules for all spacefaring nations have been put in place half a century ago (Planetary Protection Policy, maintained by the Committee on Space Research (COSPAR), in line with Article IX of the United Nations Outer Space Treaty from 1967; [3]). These rules regulate the mission’s category, depending on its target, type and purpose, and limit the acceptable microbial contamination level accordingly [3]. However, Earth is a microbial world, and even the human body carries 4 × 10^13^ microbial cells [7] that are constantly spread into our environment [8]. As a consequence, control of microbial contamination results in a substantial effort, and needs to be implemented from the beginning of a mission planning. The number and diversity of microorganisms in close vicinity of a spacecraft during AIT (assembly, integration and test) activities are dependent on a variety of factors, such as cleanroom class and architecture, the clothing and behaviour of personnel, and the cleaning and disinfection protocol of the facility [9–11].

Modern spacecraft carry extremely sensitive equipment and instruments, and therefore cannot be easily sterilised as a whole after assembly. Consequently, spacecraft parts are often sterilised before integration, and integration is performed in bioburden controlled cleanrooms, where the spacecraft is subjected to a thorough microbial contamination control. Throughout the process, the level of microbiological contamination (bioburden) is examined carefully, and detected microorganisms are catalogued.

The bioburden control of spacecraft hardware was implemented already for the Viking mission [12]. At that time, bacterial spores were considered the hardiest forms of life on Earth, and cultivation-based procedure was developed for the assessment of the bioburden of a spacecraft, aiming to detect heat-shock resistant bacterial spores [12]. These bioburden-detection standard protocols are still used by the space agencies as a standard proxy for microbial cleanliness of a spacecraft [13, 14], but are nowadays complemented by molecular, NGS-based microbial community analysis to fully assess the associated microbiota [15–18]. In addition, the ESA standard has undergone improvements such as the implementation of the Millipore Milliflex system, allowing more rapid bioburden assessment, and the validation of more efficient sampling tools [19].

The awareness that spacecraft associated microorganisms need to be investigated and catalogued in order to improve microbial detection and sterilisation for future missions has initiated numerous studies on cleanroom and spacecraft microbiology (e.g. [20, 21]). Although the largest fraction of microorganisms detectable in cleanrooms seems to be dead or present as spores [22, 23], the diversity of survival specialists is considerable. Particularly, human-associated microorganisms, including bacteria, archaea, fungi and viruses are frequently detected [10, 22, 24]. ESA and NASA have implemented public culture collections, enabling the distribution of microbial isolates, with the goal to learn as much as possible on the resistance strategies of these microbes, and how to control these amazing survivors [25, 26].

ExoMars is a two-stage program of the European Space Agency (ESA) in cooperation with Roskosmos and contributions from NASA with the goal to detect signatures of extinct or present life on Mars. The first mission of the program arrived at Mars in October 2016 and consisted of two parts: the Trace Gas Orbiter (TGO) and an entry, descent and landing demonstrator module (Schiaparelli). The instruments were intended to study the water and geochemical setting, and measure and map methane. The second part of the program is a lander and a rover with the Pasteur payload and a 2-m drill [3], to be launched in 2020.

Following the planetary protection requirements, risks of crashing and biocontamination have to stay under strict limits. For TGO the chance of crashing on Mars has to be less than 1 in 100 for the first 20 years after launch. For the Schiaparelli lander strict biological contamination constraints were applicable. To meet these constraints the ExoMars project built a new microbiologically controlled cleanroom (BCCCR) and microbiology laboratory for Schiaparelli at Thales Alenia Space, Turin, Italy. This article reports the results from four sampling campaigns in this BCCCR, before and during the Schiaparelli spacecraft was prepared for launch.

## Methods

### Cleanroom characteristics

**Fig. 1:**
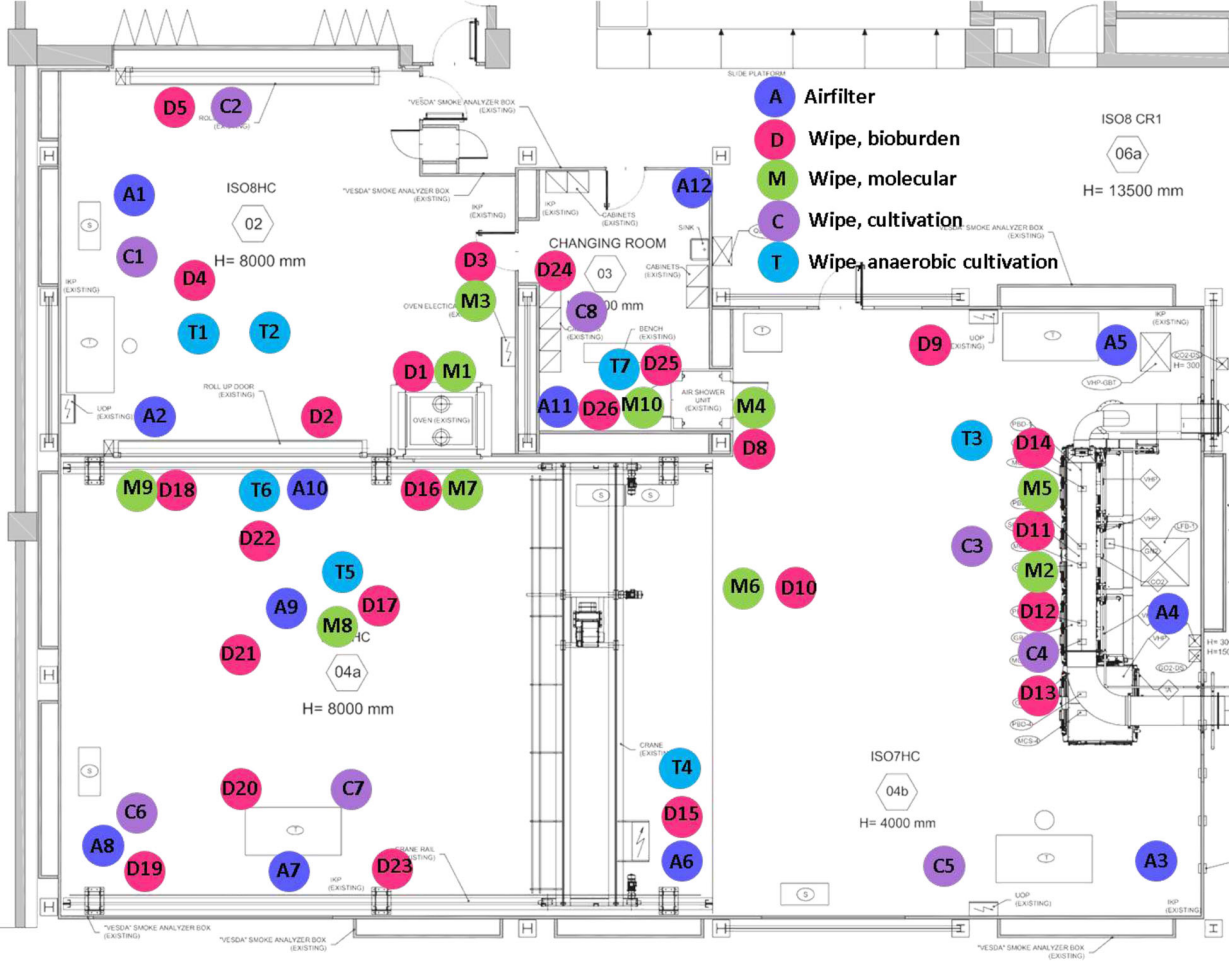
Map of the cleanroom complex in Turin (Thales Alenia) and specific sampling locations. Samples are coded as follows: *A* air sample, *D* bioburden, *M* molecular analysis, *C* aerobic cultivation, *T* anaerobic cultivation

Throughout the time frame of three years, the cleanroom complex at Thales Alenia Space in Turin, Italy, was microbiologically sampled, before the different components of the Schiaparelli hardware were moved into the cleanroom, and during the time the assembly and integration activities were performed on the Schiaparelli hardware. The microbiologically controlled cleanroom complex in Turin has been built and is maintained under the responsibility of Thales Alenia Space, Italy, in the frame of the ExoMars program. The complex is built up of three cleanrooms and one change room, where the personnel don the cleanroom garments before entering the cleanroom. The change room (room 03) is connected via air-shower to cleanroom 4b and 4a, which is separated from the cleanroom 02 with a roll-up door (Fig. 1). The cleanroom 02 is a transfer room for all equipment, and place where the cleaning appliances are stored. The change room and cleanrooms 4b and 4a are classified as microbiologically controlled ISO 7, and cleanroom 02 as microbiologically controlled ISO 8 (Fig. 1). The cleanroom classification is based on 0.5-μm-sized and larger particles with limits at 3.52 × 10^5^ for ISO 7 per m^3^ air (ISO standard for cleanrooms and associated controlled environments; https://www.iso.org/obp/ui/#iso:std:iso:14644:-1:ed-1:v1:en). The cleanliness class is reached with a suitable number of air exchanges per hour, use of HEPA (high efficiency particulate air filter) filter unit, and a rigorous cleaning routine and strict access control.

During the entire time frame, the cleanroom was cleaned daily by dusting the floor, tables, shelves, doors, handles, and walls using sterile cloths. The cleaning agent used for decontamination varied during the assembly, integration and testing period (Additional file 1: Table S1). During each cleaning cycle, the cleaning started from the cleanest area (ISO 7 HC), and continued towards the less clean change room (03). The flight hardware was covered during cleaning. All the cleaning support tools (cart, brooms and buckets) were cleaned with IPA/sporicide, and kept in the ISO 8 room. The personnel underwent medical checks and specific trainings, in order to keep the microbial contamination as low as possible.

Overall, the flight hardware underwent numerous cleaning and sterilisation cycles, including application of 70% isopropyl alcohol cleaning and dry-heat bioburden reduction. More than 3000 routine microbiological (bioburden) tests of spacecraft and cleanroom surfaces and air were performed during the assembly of Schiaparelli. Personnel was specifically trained and had to wear full body garments, including face masks and sterilised gloves, and was medically checked with respect to infections and skin diseases, and the access of people was normally restricted to four to five. During testing at Thales Alenia Space in Cannes, France, a portable “clean tent” was installed in a ISO 8 cleanroom to guarantee the microbial integrity of the spacecraft. Before launch at Baikonur on March 14, 2016, the Schiaparelli spacecraft had to be transported from Italy to Kazakhstan. The entire transport required numerous logistical pre-cautions, including double-sealing, specific air filters and a specific design of a container. In the cleanroom close to the launch site, the spacecraft was again protected from contamination using the “clean tent” for conducting further assembly steps and testing of the Schiaparelli lander.

### Sampling specifics, tools and locations

During the period of interest, the cleanroom complex was sampled four times, namely in September 18 2013 (sampling campaign 06), May 27 2014 (08), December 8 2014 (11) and February 18 2015 (12). The first sampling took place before the flight hardware were brought in for integration immediately after commissioning. All procedures are described in detail in the ECSS (European Cooperation for Space Standardisation) document ECSS-Q-ST-70-55C [13]. Samples were collected from floor (areas approx. 1 m^2^), walls and facility structures, ground support equipment (GSE) and spacecraft hardware by using gamma sterilised bonded polyester cleanroom wipes (TX3211; ITW Texwipe, Kernesville, NC, USA). Wipes for bioburden and alternative cultivation assays were, before sampling, pre-moistened with 15 ml water and autoclaved, and for cultivation of anaerobes, wipes were autoclaved dry. Wipes to be used for molecular analyses were first baked at 170 °C for 24 h (to destroy remnants of DNA), then pre-moistened with 15 ml sterile water (microfiltered at 0.2 μm; LiChrosolv, Merck Millipore), and finally autoclaved in sterile and pyrogen-free PP tubes (Sarstedt, Germany). Smaller parts of the spacecraft hardware were sampled with nylon-flocked swabs (Copan FLOQSwab 552C, COPAN, Italy). The sampling gloves for aerobic and anaerobic cultivation samples were sterilised by autoclaving, and the DNA free gloves for molecular sampling were first UV-sterilised and then autoclaved. Samples were collected by using always a new, sterile and DNA free pair of gloves for each new wipe sample. For wipe sampling, the wipe was placed flat on the surface and rubbed over the entire surface using a firm, steady pressure. The same sample area was wiped a total of three times, rotating the direction of motion first 90° and then 135°. The field negative control samples were collected by removing a sterile sampling wipe from its sterile and pyrogen-free PP tube, opening the wipe and placing it back to the tube. In laboratory, extraction blank samples were used to control the sterility of reagents and equipment. Controls were processed and analysed in the same way as samples.

Air samples were taken using a Sartorius AirPortMD8 device. Five hundred litres of air were sampled with a flow rate of 30 L/min, and the samples were collected on disposable gelatine filters [25]. Except for the spacecraft hardware the same sampling spots were sampled in each of the sampling campaigns. The sampling locations are given in Fig. 1 and described in more detail in Additional file 2: Table S2, Additional file 3: Table S3, Additional file 4: Table S4 and Additional file 5: Table S5.

Samples were taken for i) bioburden measurements (aiming at heat-shock resistant microorganisms, mostly spores), ii) biodiversity measurements (“alternative assays”, aiming at oligotrophic, alkaliphilic, mesophilic and anaerobic microorganisms as well as fungi) and iii) molecular measurements (16S rRNA gene sequencing-based microbial community analyses). For each of the procedures, different samples were taken and processed independently, including field blanks and laboratory extraction controls. All samples were transported to laboratory under cooled conditions (4–8 °C) within of 24 h before processing. Samples for cultivation of anaerobes (dry wipes) were stored at +4 °C for maximum of one week, whereas the samples for molecular analyses were immediately frozen (−20 °C) until processing.

### Bioburden assays

Bioburden assays were performed as described in ECSS-Q-ST-70-55C [13]. In brief, collected samples were extracted in sterile water (swabs) and PBS buffer incl. Tween 80 (0.02%, *w*/*v*) (wipes) by a combination of vortexing and sonication. The remaining liquid was heat-shocked at 80 °C (15 min), afterwards plated on R2A, and incubated for 72 h at 32 °C. Appearing colonies were counted after 24, 48 and 72 h.

During each sampling event, 26 wipe samples (D1-D26) from cleanrooms and change room were taken (see Additional file 2: Table S2 and Fig. 1). Five field controls, and five laboratory controls were processed in parallel (Additional file 2: Table S2). Additionally, during the second, third and fourth sampling, spacecraft hardware was sampled with swabs and wipes, as given in full detail in Additional file 2: Table S2.

### Air samples

During each sampling campaign, at least 10 air samples were taken, and at least two field controls, as well as laboratory controls were processed in parallel. Gelatine filters retrieved from the Sartorius AirPortMD8 device filter cassette were removed and immediately placed on an R2A agar plate. The colonies were counted after 72 h incubation at 32 °C. Details on sampling locations are given in Additional file 3: Table S3.

### Biodiversity assays (“alternative assays”)

Wipe samples were extracted in PBS buffer incl. Tween 80 (0.02%, *w*/*v*; PBST) by a combination of vortexing and sonication [13]. The solution was, in 20 ml aliquots, concentrated via filtration onto 0.45 μm filters (Millipore, S-Pak-Filter, 47 mm, sterile and made of hydrophilic polyvinylidene fluoride (PVDF)) under aseptic conditions. Each cultivation assay was performed in duplicates by placing the filters on (i) RAVAN agar incl. 50 μg/ml nystatin for oligotrophs [27] (modified: 1:100 diluted, final concentration of 50 mg/l sodium pyruvate instead of 20 mg/l pyruvic acid), (ii) R2A pH 9 for alkaliphiles (R2A, BD Difco; pH was adjusted with sterile Na-sesquicarbonate), (iii) PDA for fungi (potato dextrose agar, BD) and (iv) R2A for vegetative mesophiles (R2A, BD Difco), respectively. Anaerobes were cultivated on TSA (trypticase soy agar) medium, but processed under strictly anoxic conditions, as described earlier [25]. Incubations were performed at 32 °C ± 1 °C for up to 2 weeks (oligotrophs). The colonies were counted and reported at time points 24, 48 and 72 h (vegetatives and fungi), 1, 3, 5, 7 and 14 days (oligotrophs), and 1, 3, and 7 days (anaerobes). Details on the sampling locations etc. are given in Additional file 4: Table S4.

### Taxonomic analyses of the isolates by MALDI-TOF

Selected isolates obtained from four sampling campaigns were processed at the premises of DSMZ (German Collection of Microorganisms and Cell Cultures, Braunschweig, Germany) and analysed by MALDI-TOF mass spectrometry. MALDI-TOF analysis was used for grouping the strains into various clusters and to identify the isolates to species level. Colony morphology of all strains was checked from subcultures grown on agar plates. Routine media used for subculturing included R2A and TSA. Cell morphology was examined from liquid cultures by using phase-contrast microscopy (Zeiss Axioscope A.1, ×100 Plan-Neofluar oil-immersion objective, Ph3; Zeiss Axiocam MRc, and Software Axiovision). Agar slides were coated with a layer of highly purified agar (2%) and 20 μl of the freshly grown liquid culture was dropped onto the agar layer and spread by a cover slip. Aliquots from pure cultures were stored in Microbank tubes (Prolab Diagnostics) for short term storage. Pure cultures were characterised by MALDI-TOF, and when needed identified by 16S rRNA gene sequencing or automated ribotyping in addition.

MALDI-TOF sample preparation followed protocol 3 as described [28]. After ethanol–formic acid extraction, 1 μl of supernatant was transferred onto a target plate and allowed to dry in air at normal room temperature. Subsequently the sample was overlaid with 1 μl of the matrix solution and air-dried again. MALDI-TOF mass spectrometry was conducted using a Microflex L20 mass spectrometer (Bruker Daltonics) equipped with a N2 laser. Spectra were collected as a sum of 500 shots across as spot. A mass range of 2000–20,000 m/z was used for analysis. Spectra obtained for all isolates were analysed, and compared with reference spectra from the database for identification using the BioTyper software (Bruker Daltonics). Riboprinting was applied using the automated Riboprinter microbial characterisation system (Dupont, Qualicon). Sample preparation and analysis were performed according to the manufacturer’s instructions and EcoRI restriction enzyme was used to generate the DNA fragments.

### Maintenance of cleanroom isolates for long term storage

Cryopreservation in glass capillaries was used for long-term storage of chosen cleanroom isolates, which were previously selected from the MALDI-TOF groups. Freshly grown cells were harvested by centrifugation and resuspended in a small aliquot of the sterile medium (1 ml) containing a cryoprotectant. The capillaries were filled by using a micropipetting aid and subsequently flame sealed on both ends. Glycerol or dimethyl sulfoxide (DMSO) may be used as cryoprotectant. The final concentration of glycerol is usually 10–15% (*v*/v). DMSO is used at a final concentration of 5% (v/v). DSMO may be either filter sterilised or autoclaved under a nitrogen atmosphere at 115 °C for 15 min. A detailed protocol of this technique is publicly accessible at www.cabri.org. Prepared glass capillaries were placed in a storage container and laid in the gas phase of the liquid nitrogen tank first. Once frozen, the storage container was placed on its selected position in the tank.

### Molecular assays

During each sampling, 10 wipe samples for molecular analyses were taken. A list of these samples is given in Additional file 5: Table S5, and sampling locations are indicated in Fig. 1.

Due to the low biomass, three molecular samples taken from each cleanroom during each sampling campaign were pooled, as were the extraction blanks. Samples from change room and field blank were processed individually. The wipes were transferred into DNA-free bottles (baked at 250 °C for 24 h) filled with of PCR grade water. The bottles were sonicated for 120 s ± 5 s with a maximal power of 240 W and a frequency of 40 kHz, and vortexed at maximum speed for 1 min. The biomass-containing water suspension was concentrated to 200–500 μl using UV sterilised Amicon filters (Amicon Ultra 15 ml, 50 K, Merck Millipore). DNA extraction was performed using a modified XS-buffer method [24] with a bead beating step to disrupt thick-walled microorganisms such as bacterial spores, using beating tubes included in the MO BIO Power Biofilm^TM^ DNA Isolation Kit (Carlsbad, CA, USA).

For molecular cloning, near full length 16S rRNA gene was amplified using the TaKaRa Ex Taq polymerase (Clontech, Japan) with the primer 9bF [29] and 1406uR [30] under the following PCR conditions: initial denaturation at 95 °C for 2 min, followed by 30 cycles of denaturation 96 °C 30 s, annealing 60 °C 30 s, extension 72 °C 60 s, and a final extension at 72 °C 10 min. PCR products were visualised on a 1.5% agarose gel. 1 μl of each PCR product was cloned in StrataClone SoloPack competent cells (Agilent Technologies, StrataClone PCR Cloning Kit) according to manufacturer’s instructions. Depending on the concentration of the PCR product, 24–96 clones of each sample were sent to Macrogen in Amsterdam for unidirectional sequencing (primer 1406uR; [30]). In addition, the community composition and diversity of Bacteria and Archaea in ExoMars cleanrooms was studied using next generation sequencing (NGS; Illumina MiSeq). For this approach, variable region V4 of 16S rRNA gene was amplified with “universal” PCR primers 515F (5′-GTGCCAGCMGCCGCGGTAA-3′) and 806R (5′-GGACTACHVGGGTWTCTAAT-3′) [31] using TaKaRa Ex Taq polymerase (Clontech, Japan). Cycling conditions consisted of an initial denaturation at 94 °C for 3 min, followed by 35 cycles of denaturation 94 °C 45 s, annealing 50 °C 60 s, extension 72 °C 90 s, and a final extension at 72 °C 10 min. The produced fragments were sequenced at ZMF Core Facility Molecular Biology in Graz, Austria, using the available Illumina MiSeq platform.

### Sequence data analysis of cloning and next generation sequencing

To analyse the microbial community composition and taxonomic diversity obtained raw reads were processed using mothur version 1.36.1 [32] following the Standard Operation Procedure (SOP): For MiSeq data, the paired end reads were joined together, and the produced sequences were quality checked (minimum length 200, maximum length 300, maximum number of homopolymers 8) and aligned against mothur formatted SILVA 123 database [33]. Then, the good quality sequences were pre-clustered and chimeric sequences were removed. The number of sequences per sample before and after quality control was following: September 13 change room: before qc 61,610/after qc 40,831, September 13 cleanroom 4a: 47,961/30141, September 13 cleanroom 02: 49,332/30093, December 14 change room: 47,036/30127, December 14 cleanroom 4a: 55,643/38875, December 14 cleanroom 02: 60,188/38046, February 15 change room: 50,252/33269, February 15 cleanroom 4a: 76,842/39656. Taxonomic assignment was performed by querying the sequence reads against a trainset14_032015 reference database, and the sequences were clustered into OTUs (threshold 0.03 dissimilarity) using average neighbour algorithm. A biom table was constructed for downstream analyses, and OTUs represented by 5 or less sequences were removed. The Sanger data was processed similarly, except for merging the raw reads and quality check in the beginning, as the raw data was received in fasta format. These data processing steps were performed in Galaxy, which is an open source web-based platform for data processing and analysis [34]. This platform was made available by the Center for Medical Research (ZMF), Medical University of Graz. To further analyse these datasets, to calculate alpha and beta diversities, differences in community composition, and visualise the results, we applied Calypso (Version 5.8), an online platform for mining, visualising and comparing multiple microbial community composition data (cgenome.net/calypso). Total-sum normalisation was applied for 16S rRNA gene data. For network and functional analyses we only used the MiSeq data. To create the networks, OTUs were clustered and weights were calculated using a stochastic spring-embedded algorithm. The resulting edge and node tables were visualised using Cytoscape 2.8.3 [35]. Here, the OTUs were coloured by their sample origin and their relative abundance was correlated with the node size. To analyse the predicted functions of studied microbial communities, the sequence data was processed with Qiime [36] open-reference OTU picking pipeline with GreenGenes taxonomy (13_8 database [37]), and a PICRUSt (version 1.0.0.) analysis was performed using the default settings [38]. Text formatted biom tables of MiSeq and Sanger sequence data are given in the Additional file 6: Table S6 and Additional file 7: Table S7. Details about data analysis pipelines are given in Additional file 8. Sequence data were submitted to the European Nucleotide Archive (ENA) with the study accession number PRJEB15908.

### Quantitative real-time PCR

Ribosomal RNA gene copy numbers were quantified with quantitative real-time PCR (qPCR) in triplicates using the RotorGene 6000 Real-Time PCR system (Corbett Life Science, Concorde, NSW, Australia). The qPCR reactions were carried out in volume of 10 μl: 5 μl 1 x SYBR Green Taq Premix (Quantitect SYBR Green PCR kit, Qiagen, Hilden, Germany), 1 μl of each primer (3 μM), 2 μl water (Merck Millipore), and 1 μl of extracted DNA template. To quantify Bacteria we applied Bacteria specific primers 338bF and 517uR [30]. Purified standard of *Desulfovibrio desulfuricans* and negative controls (no template) were included in all qPCR runs. The quantification of standard was performed with the Qubit Quantitation Platform 2.0 (High Sensitivity Kit, Invitrogen, Carlsbad, CA, USA). Cycling conditions for qPCR consisted of an initial denaturation at 95 °C for 15 min and a cycling protocol as follows: denaturation at 94 °C for 15 s, annealing at 60 °C for 30 s and elongation at 72 °C for 30 s. Melting curve was generated at 72–95 °C. Copies detected in qPCR negative controls were subtracted from sample values.

## Results

### Bioburden assays reflect the consequent reduction of microbial contamination throughout the sampling period, reaching the detection limit at the last sampling

**Fig. 2:**
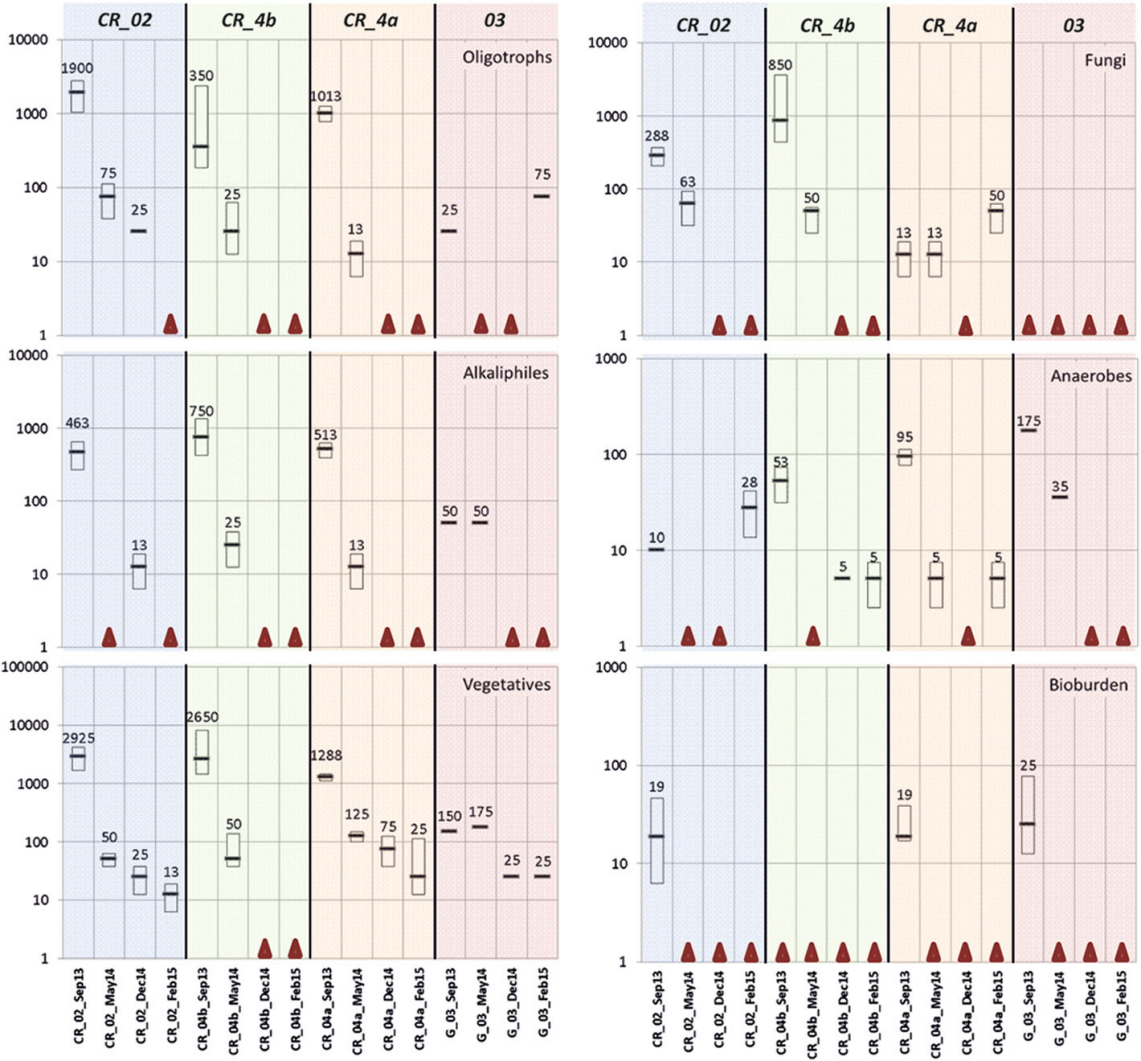
Retrieved colony forming units from alternative assays (oligotrophs, alkaliphiles, vegetatives, fungi and anaerobes) and bioburden measurements, grouped by sample origin. Horizontal lines and given numbers reflect the median of all samples taken at this certain timepoint in the respective cleanroom (source: Additional file 2: Table S2 and Additional file 4: Table S4). Boxes represent the first and third quartile. Red triangles refer to a median of 0. Colony forming units (CFUs) are given in a logarithmic scale (*Y*-axis). Colours indicate the different sampling campaigns, sampling locations are indicated (*X*-axis)

The bioburden assays aimed at the detection of heat-shock resistant microorganisms that are considered to represent the most harmful microbial contamination source for planetary protection issues. In detail, the median of colony forming units (CFUs) in cleanrooms 02, 4a, 4b and change room 03, was zero throughout the time the spacecraft hardware was in the facility (Fig. 2). No colonies were retrieved from samples taken during the February 2015 (last) sampling and thus our methods reached the detection limit. Before AIT activities started, the median of the bioburden was higher in cleanrooms 02 and 4a, as well as in the garment room 03 (19, 19, and 25 CFUs per m^2^, respectively) (Fig. 2, Additional file 2: Table S2). The strict cleaning and management regime of the cleanroom complex was found to result in a substantial reduction of the bioburden. Swab and wipe samples taken from spacecraft hardware revealed zero CFUs for the last sampling and extremely low counts for the other two samplings before (Additional file 2: Table S2). Only a few microorganisms were retrieved from heat-shocked samples taken from spacecraft hardware.

### Overall cultivable diversity decreased during AIT activities

The biodiversity assays aimed at the cultivation of a broader diversity of microorganisms from the cleanroom facility, including oligotrophs, alkaliphiles, vegetatives, fungi and anaerobes [18, 23, 25, 39].

The highest median CFU counts were observed for vegetative microorganisms, which remained detectable also during the last sampling event (Additional file 4: Table S4, Fig. 2). However, the microbial contamination load was substantially reduced compared to the first sampling, when spacecraft hardware had not entered the cleanrooms yet. Notably, no CFUs were observed on PDA agar (‘Fungi’) within the change room (room 03), throughout the sampling period, although bacteria were found to be present therein. Overall, the OTUs retrieved from alternative assays (as well from air samples, Additional file 3: Table S3), revealed a decreased amount of cultivable microorganisms in the course of AIT activities, confirming the increased microbial cleanliness observed by bioburden measurements.

### MALDI-TOF mass spectrometry allowed the reliable identification of microbial isolates

A total of 113 isolates originally obtained during the sampling period were analysed by MALDI-TOF mass spectrometry. In case that identification of a cleanroom isolate failed using MALDI-TOF, characterisation was completed by 16S rRNA gene sequencing or automated ribotyping. A summary of all isolates data is shown in Additional file 9: Table S8, and a dendrogram of identified isolates is given in Additional file 10: Figure S1. Selected isolates were assigned a DSM number and are now available through the ESA catalogue at DSMZ (https://www.dsmz.de/research/microorganisms/projects/european-space-agency-microbial-strain-collection.html).

*Arthrobacter*, *Bacillus*, *Micrococcus* and *Staphylococcus* were the most abundant isolates obtained and processed (Additional file 9: Table S8). Whereas *Bacillus* and *Staphylococcus* species were obtained from a number of surfaces and under different enrichment conditions, *Arthrobacter* was detected during the first sampling only, and solely on cleanroom surfaces. Within the bioburden studies (see Additional file 11: Table S9.) endospore forming bacteria *Bacillus mycoides, B. subtilis* and *B. megaterium* where the only retrieved contaminants from the cleanroom areas during all sampling campaigns, and only few microorganisms were retrieved from heat-shocked samples taken from spacecraft hardware, namely *Staphylococcus pasteuri*, *S. warneri*, *S.* sp., *Rothia amarae*, *B. subtilis* and *B. mycoides* (Additional file 11: Table S9). The *Staphylococcus* species are clearly associated with the human body as its source. *B. mycoides* and *B. subtilis* were the only spore-forming microorganisms retrieved from spacecraft hardware. Interestingly, isolates enriched in September 2013 and December 2014 were found to be related to the *Bacillus subtilis* group, but strains obtained in February or May prevalently to *B. mycoides*, *B. pumilus*, or *B. simplex* (Additional file 11: Table S9). Although the *B. mycoides* strains were isolated from different locations, the riboprint pattern obtained for seven of the isolates could be assigned to the same ribogroup, indicating that one strain might be spread out within cleanroom 4b (Additional file 12: Figure S2).

The highest diversity in general was detectable within the samplings obtained in September 2013 (19 genera), followed by samples received in May 2014 (8 genera). Sixteen bacterial genera were cultivated on R2A agar from sampling campaign in September 2013 (*Agrococcus, Arthrobacter, Bacillus, Brevibacterium, Brevundimonas, Cryptococcus (yeast), Hymenobacter, Kocuria, Massilia, Methylobacterium, Paenibacillus, Paracoccus, Pedobacter, Rathayibacter, Rhodosporidium, Staphylococcus*), whereas only 6 of these, namely *Bacillus, Kocuria, Methylobacterium, Paenibacillus, Paracoccus* and *Staphylococcus* species were observed during AIT activities. Isolates related to *Arthrobacter* and *Hymenobacter* could only be detected in samples taken in September 2013, and *Methylobacterium* and *Kocuria* occurred in May 2014. *Micrococcus luteus* and *Paracoccus yeei* were predominant in February 2015. Staphylococci were detectable in all samplings, but mostly in September 2013, May and December 2014. 12 isolates were identified as *Staphylococcus hominis* and three isolates as *S. warneri*. Only a few isolates could been assigned to the species *S. aureus*, *S. epidermidis*, *S. saprophyticus*, *S. lugdunensis* or *S. simulans*.

*Micrococcus* was obtained during three of four sampling events, and was mainly detected on cleanroom surfaces. Both species, *Arthrobacter* and *Micrococcus*, preferred specific enrichment media, namely oligotrophic and alkaliphilic conditions. Those microbes, as many others, where not detected when a heatshock was applied to the sample (bioburden assays).

Fungal genera, namely *Trichoderma, Gibellulopsis, Alternaria* and *Lecythophora*, were only found in cleanroom 02 in September 2013, whereas different yeast species (see Additional file 11: Table S9) were found in cleanrooms 02 and 04b. A broad microbial diversity was retrieved from RAVAN agar, targeting microorganisms that can grow under extreme nutrient constraints, including *Kocuria, Micrococcus, Microbacterium, Arthrobacter, Rathayibacter, Curtobacterium* (all Actinobacteria), *Bacillus* (Firmicutes), *Paracoccus, Methylobacterium, Roseomonas, Sphingomonas* (all α-Proteobacteria), *Hymenobacter* (Bacteroidetes) and *Pigmentiphaga* (β-Proteobacteria). Strictly and facultatively anaerobic microorganisms were enriched on TSA plates that were incubated under anoxic conditions. In particular, *Staphylococcus*, *Bacillus*, and *Dermabacter* species were enriched by this method, representing facultatively anaerobic bacteria. During the last, cleanest sampling, only *Bacillus*, *Micrococcus*, *Paracoccus* (three strains) and *Staphylococcus* were grown.

### Results from quantitative PCR indicate the presence of DNA signatures from dead cells in the cleanroom areas

Although bioburden and alternative measurements revealed a subsequent reduction of the microbial contamination, in particular during AIT activities, the results from quantitative PCR were less informative. The lowest contamination with 16S rRNA gene copies was found in cleanroom 4b throughout the analysed time frame (range from below the detection limit (BDL) to 23.204 copies/m^2^). However, the clear trend of decreasing microbial load could neither be followed in the cleanrooms (range from BDL to 49.477 copies/m^2^), nor in the change room (range from BDL to 99.690 copies/m^2^) (Additional file 13: Figure S3).

### Sanger sequencing results display a strong impact of human associated bacteria on cleanroom microbiota

**Fig. 3:**
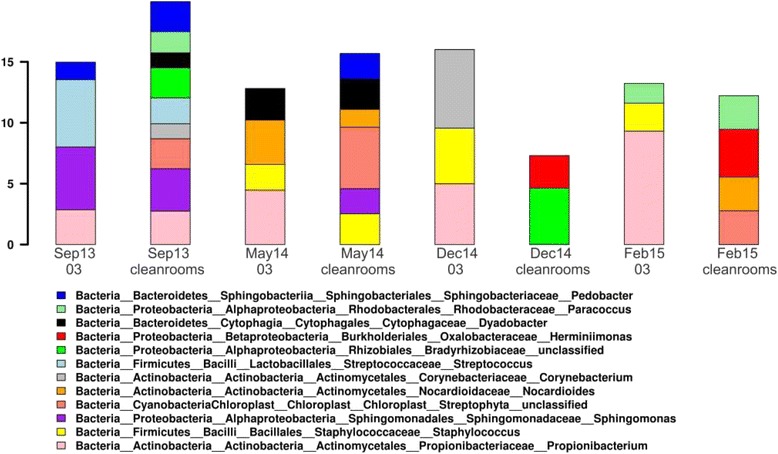
Barchart depicting the 20 most abundant taxa in Sanger sequencing data. Cleanroom samples from each sampling campaign were pooled together to obtain a signal and marked as ‘cleanrooms’, and change room samples were marked with the room number ‘03’

We also studied the cleanroom microbial communities using Sanger sequencing, following cloning of the 16S rRNA gene pool. In total, we identified 70 different bacterial taxa. Several human skin associated bacteria were abundant in the dataset: *Propionibacterium* was the most abundant genus, and always present in change rooms, as well as in cleanrooms in September 2013, before the AIT activities started. *Propionibacterium* was also the only genus that was statistically significantly increased in change room compared to cleanrooms (analysis of variance, *p* ≤ 0.05, rank test, *p* = 0.027). Additionally, other human and human skin associated bacteria, such as *Streptococcus*, *Staphylococcus* and *Corynebacterium* tended to be more abundant or solely present in the change room. Additionally, *Streptococcus* and *Sphingomonas* were more abundant before the AIT activities started (Bayesian analysis of variance, *p* ≤ 0.05). At all sampling campaigns, cleanrooms carried more diverse bacterial communities compared to change room (Fisher’s alpha index, *p* = 0.01), and the cleanrooms were most rich in microbial diversity before the AIT activities started, further strengthening the observation of decreasing contamination towards the end of project. The 20 most abundant bacterial genera in Turin cleanrooms based on cloning and Sanger sequencing are presented in Fig. 3.

### Next generation sequencing uncovers the diversity and divergence of cleanroom microbial communities

We further characterised the microbial communities in ExoMars cleanrooms using Illumina MiSeq next generation sequencing. In the whole dataset, we identified 15 microbial phyla, of which 12 were affiliated to Bacteria and 3 to Archaea (Thaumarchaeota/*Nitrososphaera*, Euryarchaeota/*Pyrococcus* and unclassified Archaea). The most abundant bacterial phyla were Proteobacteria (76% of all sequence reads), Firmicutes (11%), Actinobacteria (10%), and Bacteroidetes (2%). The most abundant identified genera were *Cupriavidus* (41% of all sequence reads), *Pseudomonas* (10%) *Staphylococcus* (7%), *Corynebacterium* (6%), and *Delftia* (3%). *Sphingomonas* and *Acinetobacter* were detected at 1% relative abundance. All other taxa were represented by less than 1% of the total sequence reads.

The diversity of cleanroom microbial communities changed in the course of the project. On the first sampling campaign, before the start of AIT activities, microbial α-diversity in cleanrooms was relatively high (inverse simpson index 24–26). The change room carried considerably less diversity, and after changing the cleaning solutions before the next sampling campaigns, the diversity also decreased in cleanrooms and stayed below the approved limit throughout the project.

**Fig. 4:**
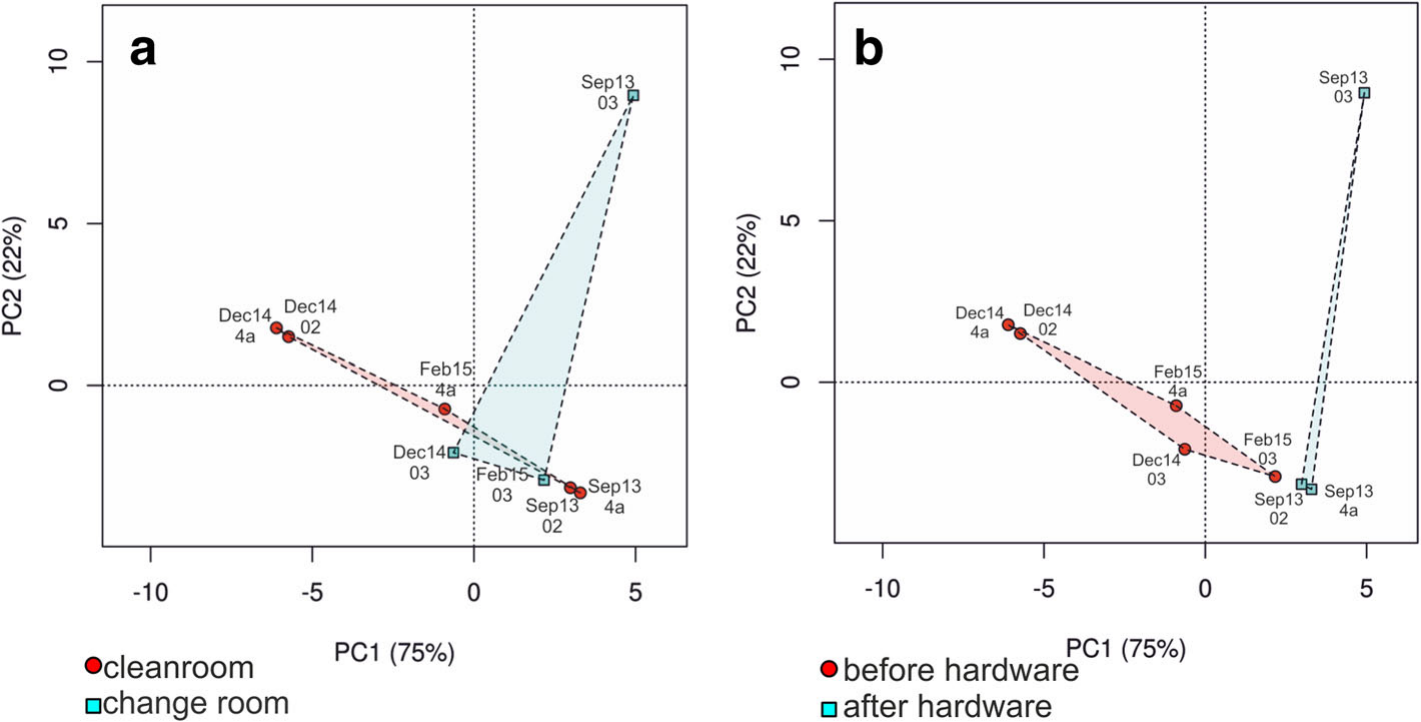
PCA plot depicting the relationships of cleanroom and change room microbial communities (**a**) and communities before and after the spacecraft hardware was brought in (**b**) based on taxonomy information

We also compared the community structures of cleanrooms and change room at different sampling time points (β-diversity). Cleanrooms and change room carried distinct microbial communities: principal component analysis (Fig. 4) demonstrates that especially in the beginning of the study in September 2013, the change room groups far from other sampling time points and locations, indicating a distinct community structure. The cleanroom samples collected on the same day were always highly similar and grouped together, and the samples gathered in separate time points were always different to certain extent. Furthermore, the principal component analysis comparing the microbial communities before and after the AIT activities started shows that there is a shift in the community structure after the hardware were brought in the cleanroom and the cleaning regime changed, suggesting that the microbial community is determined by external factors, such as the work conducted in cleanroom, personnel, and cleaning regime.

The identified taxa were also compared with the linear discriminant analysis (LDA) effect size method (LEfSe) [40] to find those taxa that are significantly different between the compared sample groups. Human skin associated *Staphylococcus* and a soil bacterium *Mucilaginibacter* were more abundant before the AIT activities started, and *Cupriavidus*, the highly resistant bacterium, was more abundant after the hardware were brought in and the cleaning regime changed to more efficient (LEfSe, *p* ≤ 0.05).

### Predicted functions indicate an elevated stress level for microbial inhabitants during AIT activities

We applied PICRUSt [38] to predict the microbial genes present in the ExoMars cleanroom based on MiSeq amplicon data. In general, the most abundant identified functions were involved in membrane functions, such as transport systems and receptor proteins, quorum sensing and environmental information processing.

We compared the community functions between cleanrooms and change room, and before and after the start of AIT activities in order to assess if the level of confinement and mode of use affected the predicted community function. Principal components analysis plots visualise the distinct communities between cleanrooms and change room throughout the the ExoMars assembly, and between the first sampling and after the hardware were in the cleanrooms (Additional file 14: Figure S4).

The identified functions were also compared with the linear discriminant analysis (LDA) effect size method (LEfSe) [40] to find the most differentially abundant functions between cleanrooms and change room and before and after AIT activities were initiated. Several predicted genes were more abundant in change room, including genes coding for base excision repair protein exodeoxyribonuclease, and several genes involved in membrane functions. In cleanrooms, motility associated genes, such as pilus assembly protein and chemosensory two-component regulatory system were more abundant.

We also studied how the initiation of AIT activities and change in cleaning regime affected the microbial communities at functional level. The results show that functions related to nucleotide excision repair, ABC transporters, quorum sensing, and metabolism of terpenoids and polyketides, as well as biodegradation and metabolism xenobiotics increased in relative abundance after the activities started and the cleanroom was cleaned with more effective cleaning products.

### AIT activities have tremendous impact on the microbial community composition and the microbial transfer from one room to another

**Fig. 5:**
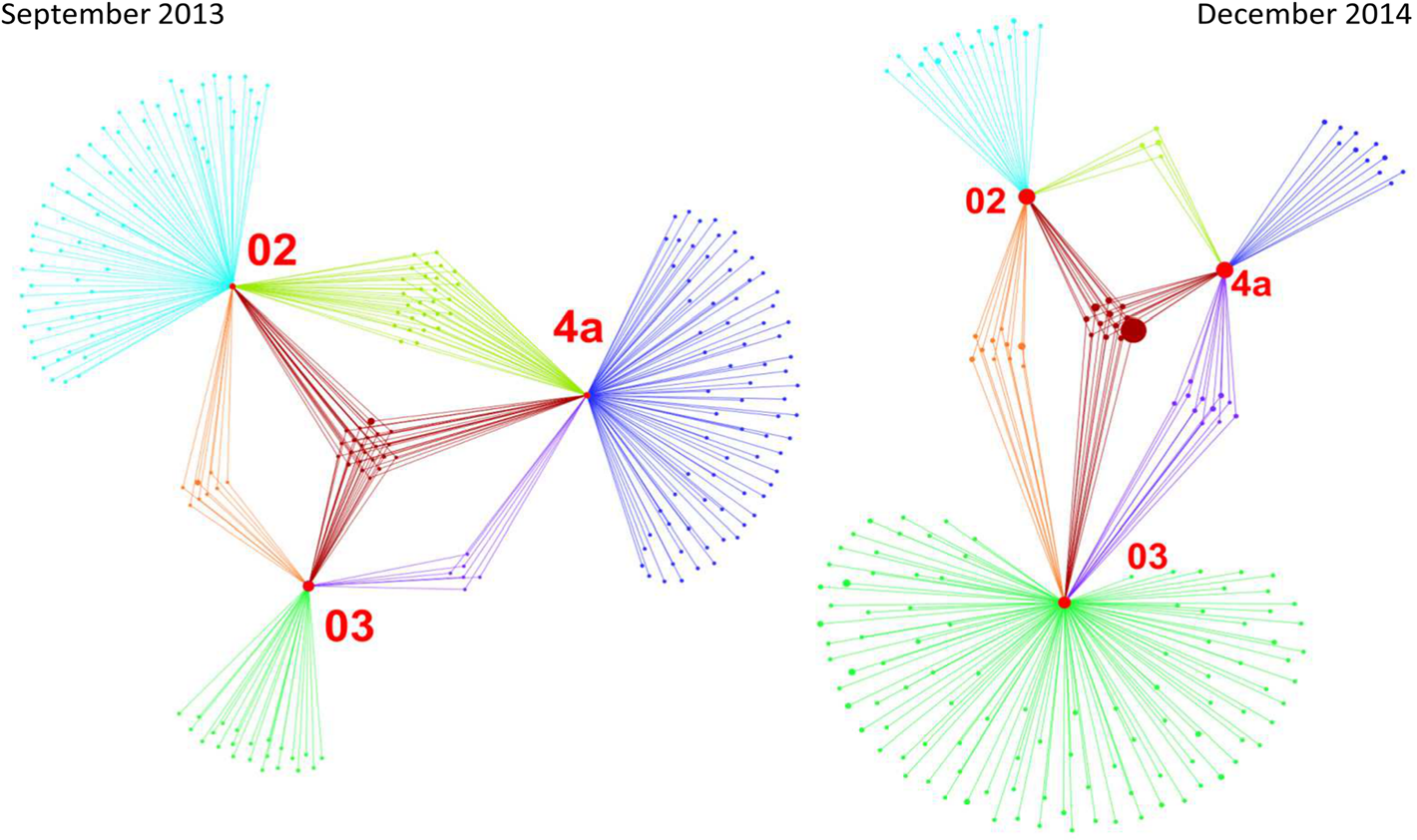
Cytoscape network for two representative sampling events: September 2013 (before AIT) and December 2014 (cleanroom harboured spacecraft hardware). 02 and 4a refer to cleanroom areas, whereas 03 is the changing room. A more detailed picture of the two networks, including taxonomies, is given in Additional file 15: Figure S5 and Additional file 16: Figure S6

The two representative network analyses (before and during the cleanroom complex harboured spacecraft hardware) show a different picture of the microbial community in the single rooms and the overlap of the found taxonomies. Notably, the change room harbours a less unique microbial community during the first sampling in September 2013, whereas the cleanroom areas 02 and 4a reveal quite a number of microbial signatures unique to their location. The picture is drastically different in December 2014, where the change room harbours the largest number of unique taxons (Fig. 5; Additional file 15: Figure S5 and Additional file 16: Figure S6). The core microbiota of all three areas was composed of signatures from, e.g. *Pelomonas, Sphingomonas, Methylobacterium, Pseudomonas, Cupriavidus, Escherichia/Shigella, Micrococcus, Brevundimonas, Corynebacterium, Delftia, Acinetobacter, Staphylococcus*, *Enhydrobacter* (September 2013), and *Delftia, Micrococcus, Staphylococcus, Rhizobium, Cupriavidus, Escherichia/Shigella, Enhydrobacter,* and *Sphingomonas* (December 2014), with those printed in bold letters appearing in both core microbiotas (Additional file 15: Figure S5 and Additional file 16: Figure S6). *Cupriavidus* was the most prominent taxon found in the core microbiota of the December 2014 sampling. During December 2014, only four microbial taxa were found to be distributed from cleanroom 02 and 4a (besides the core taxa) and vice versa, namely unclassified Sphingomonades, *Massilia*, *Pseudomonas* and unclassified Betaproteobacteria. All in all, the network analyses confirmed the extraordinary efficiency of the cleanliness regimes during the AIT activities and the presence of the spacecraft hardware in the rooms.

### Variety of methods generates unique information on the cleanroom microbial communities

**Fig. 6:**
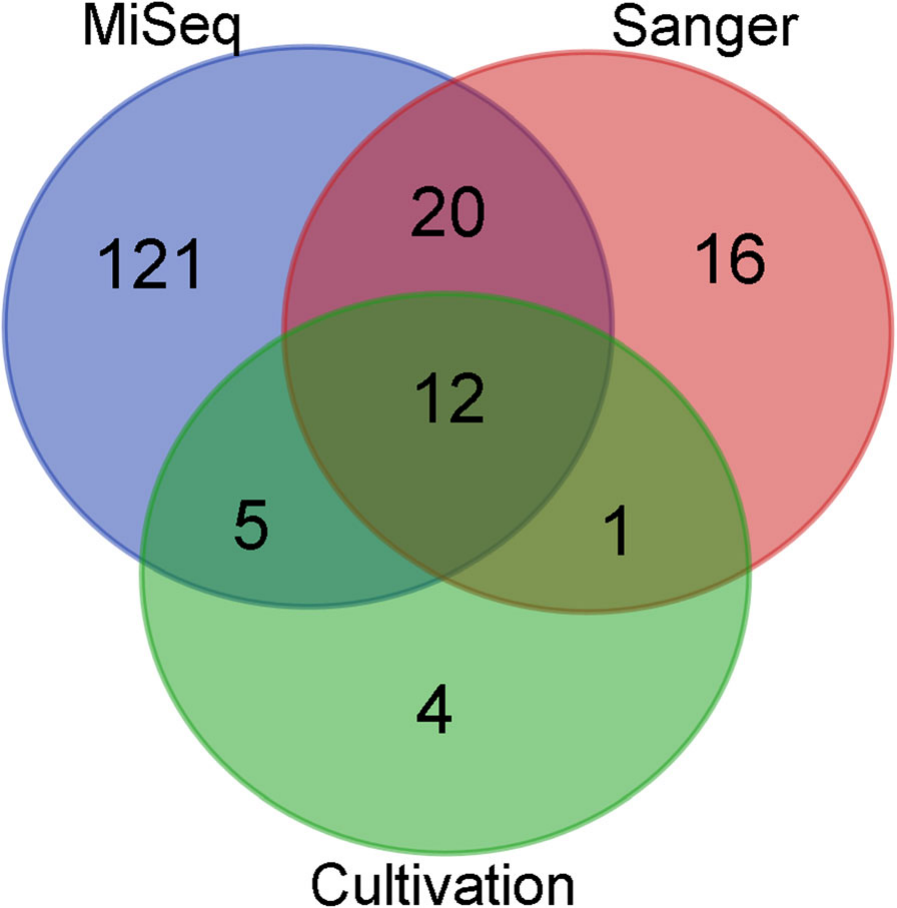
Venn diagram visualising the overlap between identified microbial genera detected in this study by cultivation, MiSeq and Sanger sequencing

We analysed the microbial community structure in ExoMars cleanrooms using three methods: MiSeq amplicon sequencing, cloning and Sanger sequencing, and cultivation. We expected merely partially overlapping results as the applied methods differ considerably. Overall, 179 different bacterial genera were detected during the entire study. With high throughput MiSeq amplicon sequencing we detected majority of these taxa (88%), but both Sanger sequencing and cultivation produced unique information on the cleanroom microbial communities. *Pigmentiphaga, Dermabacter, Luteimonas*, and *Agrococcus* were detected only via cultivation, and 16 different taxa via Sanger sequencing, inluding *Brochothrix, Oligotropha, Gemmatimonas, Dehalobacter, Pelomonas, Pseudoclavibacter, Variovorax, Parabacteroides,* and *Lactococcus*. Only 12 taxa (7%) were identified with all three methods: *Brevundimonas, Micrococcus, Staphylococcus, Pedobacter, Microbacterium, Massilia, Bacillus, Sphingomonas, Paenibacillus, Methylobacterium, Hymenobacter*, and *Paracoccus*. Venn diagram (Fig. 6) depicts the overlap of identified bacterial genera with used analysis methods. List of all detected taxa and analysis methods can be found in Additional file 2: Table S2, Additional file 3: Table S3, Additional file 4: Table S4, Additional file 5: Table S5, Additional file 6: Table S6, Additional file 7: Table S7, Additional file 9: Table S8 and Additional file 11: Table S9.

## Discussion

ESA’s ExoMars 2016 mission reached Mars in the middle of October 2016. The Schiaparelli module touched down on October 19 2016 on Meridiani Planum, a flat, even region on Mars. Due to planetary protection constraints, the spacecraft hardware was assembled under biological contamination control in specifically designed and built cleanrooms. The results confirm that the bioburden, detected microbial contamination, and microbial diversity in cleanrooms decreased during the assembly, integration and testing period after the cleanroom was decontaminated with highly effective cleaning agents and alternating pH. At no point, the spacecraft hardware showed microbial contamination levels above the expected and acceptable limit.

For the first time, the isolated microorganisms from cleanrooms and spacecraft hardware were identified using MALDI-TOF, which was found to be a very powerful tool for taxonomic determination of planetary protection-associated microbial isolates. MALDI-TOF analysis of bacterial cells is well-recognised as a fast and reliable tool for rapid characterisation and identification of bacteria, because the spectra obtained from the vegetative bacterial cells are dominated by peaks of the ribosomal proteins. These proteins are conservative molecules and best suited for classification and identification of bacterial species. As shown by Schumann and Meyer [28] changes in the cell architecture of endospore-forming bacteria, caused by sporulation, may significantly alter the MALDI-TOF mass spectra by detection of spore proteins. As a consequence, the successful identification of *Bacillus* and related species by MALDI-TOF requires that the culture under question is analysed in the same physiological state for which the database entry was generated. Following this requirement, reliable protein based differentiation of *Bacillus* isolates by MALDI-TOF was exemplary shown for the *Bacillus pumilus* [41, 42]. In our study, the identity of *Bacillus* isolates was additionally confirmed via riboprinting and 16S rRNA gene sequencing. Our results from the bioburden assays performed for the ExoMars 2016 mission were in accordance with the results retrieved from 3000 independent samples, analysed by the microbiology laboratory run by Thales Alenia Space in Turin.

A number of representatives of microorganisms, that were isolated also during this study, including *B. cereus* [43]*, B. megaterium* [44]*, B. mycoides* [45]*, B. pumilus* [46]*, B. subtilis* [47–51]*, Micrococcus luteus* [52] *and Staphylococcus aureus* [53] have been tested for the potential to survive space flight, or their capability to survive under simulated Mars conditions. In particular, *B. subtilis* (spores) has been found to be able to survive spaceflight, including impact, planetary ejection and atmospheric re-entry. *B. mycoides* is typically found in soil [54], but has been found to survive Martian simulations. Notable, *B. mycoides* isolates were retrieved several times during this study, and riboprinting indicated that the *B. mycoides* isolates (May 2014) belonged to the same ribogroup, supporting the assumption, that one strain was spread in the cleanroom 4b.

Bioburden assays, aiming to detect heat-shock surviving microorganisms did not only detect spore-forming microbes that are considered to represent the most harmful microbial contamination source for planetary protection issues, but also non-spore formers: we additionally retrieved, e.g. seven different *Staphylococcus* species from different areas. *Staphylococcus* is a typical human-associated bacterium, as were many other isolates identified in this study. Also a large proportion of bacterial signatures detected by molecular approaches were associated to the human body. We detected for example *Propionibacterium*, an anaerobic to microaerophilic chemo-organotrophic bacterium which lives in sweat glands, sebaceous glands, and other areas of the human skin, to be more abundant in the change room compared to cleanroom. Additionally, *Streptococcus*, *Staphylococcus* and *Corynebacterium,* belonging to the human microbiome, living in mouth, intestine, upper respiratory tract, and on skin, were abundant particularly in the change room which reflects the human influence in the garment area microbiota. Although the human influence, and thus the spreading of human-associated microorganisms is limited to a minimum, these microorganisms are the most abundant contaminants in cleanrooms, similarly as in other confined environments [9]. Additionally, we found environmental bacteria, including *Cupriavidus*, a chemoorganotrophic/chemolithotrophic, metal-resistant bacterium adapted to survive in harsh conditions, a strictly aerobic *Delftia*, which can cause human infections, as well as *Acinetobacter*, an environmental bacterium that can also reside on the human skin.

A number of microorganisms, including *Microbacterium, Bacillus, Methylobacterium, Hymenobacter* and *Paracoccus* were detected with all methods used in this study. *Hymenobacter* is often associated with cold environments [55, 56] and *Microbacterium* has been isolated from human clinical samples [57]. These bacteria were identified solely in first sampling. Soil and plant associated *Methylobacterium* [58] was identified in first and second sampling, and spore-forming *Bacillus* recurrently in all samplings. Additionally, *Paracoccus yeei* was found frequently in two sampling events, and in particular during the last sampling. *P. yeei* is known as a typical environmental microorganism, thriving in soil and brines, but has also been associated with unusual (skin) and eye infections in the human body [49, 50]. Interestingly, representatives of all these genera have recently been identified as highly resistant against UV-C radiation and desiccation [59] which could explain their success also in the cleanroom. Successful isolation of all these bacteria suggests that they are able to endure or even thrive in the harsh cleanroom conditions, which are known to pose extreme stress and selection pressure on the microbial inhabitants, not only due to strict cleaning and decontamination procedures, but also the lack of water, nutrients and cofactors, as has been indicated in previous studies of cleanroom associated microbial communities [10, 22, 60]. As a consequence, although the number of microorganisms is tremendously reduced by frequent cleaning procedures and decontamination, the remaining bacteria are typically survival specialists with various resistances against one or several stressors, including antibiotics, metals, radiation, desiccation, or starvation. Interestingly, most of our cultivated bacteria, namely genera *Arthrobacter, Bacillus, Brevibacterium, Brevundimonas, Curtobacterium, Kocuria, Massilia, Methylobacterium, Micrococcus, Microbacterium, Paenibacillus, Paracoccus, Pedobacter, Roseomonas* and *Sphingomonas* have previously been identified as contaminants in sequence-based microbiota analyses [61]. This finding indicates that the DNA contamination of kits or laboratory reagents may be caused by resistant bacteria that can survive in extremely severe environments.

Overall, all cultivation methods applied in this study indicated a very clear trend during the AIT activities towards a reduction of the microbial contamination under detection limit (bioburden assays) or a very low level (biodiversity assays). These findings were confirmed by molecular analyses, including cloning/Sanger sequencing, and next generation sequencing of the microbial 16S rRNA genes. A clear difference of the cleanroom microbial community was detected comparing samples from before and during hardware assembly, which was also indicated by a completely different picture of the network and microbial community spreading. The network analysis also revealed that during AIT activities, the cleaning and decontamination procedures reduced the microbial diversity in cleanroom to a very low level, even though the diversity in change room increased drastically, most likely due to high level of utilisation.

NGS data retrieved from the samples taken during AIT activities showed a lower diversity of microbial community signatures in the cleanrooms, compared to the change room. This is fairly contradictory to the statements made earlier suggesting that cleanrooms carry as diverse microbial communities as the surrounding uncontrolled adjoining facility, based on analyses of a NASA maintained cleanroom [16]. However, it shall be emphasised that the BCCC in Turin was constructed to allow a two-step gowning procedure, where staff changes their own clothes to cleanroom underwear right at the entrance of the complex, and don their cleanroom garment under cleanroom conditions (ISO 7) to reduce the microbial contamination from the change room. This two-step procedure was proposed earlier, since usually the change rooms are the most critical contamination source for cleanrooms [18].

In this study, we used a variety of methods to determine microbiological contamination of the flight hardware and cleanrooms, and performed various alternative cultivation assays, and utilised molecular techniques, including qPCR and next generation sequencing, to assess the quantity and identities of bacterial and archaeal signatures in the cleanrooms. In preparation for the next ESA mission ExoMars 2020, we will use these lessons learned for further optimization of the methodologies. Overall, here we emphasised again the importance of the so-called alternative cultivation assays, for the detection of a broader cultivable diversity, as many bacteria, including several taxa with known resistances, were only detected when a variety of different (special) cultivation media was provided, and the heatshock was not applied prior to cultivation. In addition, the superiority of the NGS-based molecular methods was clearly shown, and with the next mission ExoMars 2020 the cloning and Sanger sequencing method will not be applied any longer, as it will be fully replaced by Illumina MiSeq amplicon sequencing. Notable, the quantitative PCR method did not provide us reasonable information on the absolute microbial abundance during the study, due to the highly sensitive detection of signatures from dead and disrupted microorganisms, whereas the bioburden and alternative assays focus on the cultivable portion of the microbial load. The most informative information was retrieved from NGS analyses, and combination of bioburden and alternative assays: Bioburden assays to determine a proxy for the microbiological contamination of the flight hardware and cleanrooms, and alternative cultivation assays to reveal the cultivable biodiversity of the cleanrooms will be utilised in similar manner also the future.

## Conclusion

To conclude, we would like to highlight the importance of two-stage gowning process and highly effective cleaning and decontamination regime with alternating pH levels to reduce the bioburden, detected microbial contamination, and microbial diversity during the assembly, integration and testing activities. During this project, the spacecraft hardware never showed microbial contamination levels above the acceptable limit. Consequently, the cleanroom complex at Thales Alenia Space in Turin is an excellent example of how efficient microbiological control for flight hardware under strict planetary protection constraints can be performed in order to ensure mission success of ongoing and upcoming life detection missions.

## Additional files

Additional file 1: Table S1. Cleaning agents in Turin cleanroom. (XLSX 11 kb)

Additional file 2: Table S2. Bioburden analysis of cleanroom and spacecraft hardware samples: D: cleanroom wipe samples. The number of colony forming units (CFU) of heat-shock surviving microorganisms is given. CFU total reflects the number of CFU observed on the agar plates. Pour fraction 0.8 refers to the application of a correction factor, since only 80% of the entire sample was plated. Wipe efficiency 0.2 adds another correction, due to the efficiency of wipe sampling to recover spores from surfaces of about 20%. (XLSX 24 kb)

Additional file 3: Table S3. Overview on the air samples taken during the four sampling campaigns and the measured microbial contamination level per m^3. (XLSX 10 kb)

Additional file 4: Table S4. Biodiversity assay (“alternative assays”): Number of colony forming units (CFUs) per m^2. Numbers have been corrected by pour fraction and wipe efficiency (see Additional file 1: Table S1). (XLSX 12 kb)

Additional file 5: Table S5. Sampling locations for molecular analyses (wipe samples). (XLSX 9 kb)

Additional file 6: Table S6. Txt formatted biom table of MiSeq amplicon data. (TXT 102 kb)

Additional file 7: Table S7. Txt formatted biom table of Sanger sequencing data. (TXT 15 kb)

Additional file 8: Sequence data analysis pipelines. (DOCX 49 kb)

Additional file 9: Table S8. Retrieved microbial isolates, the sampling date, location and enrichment strategy (“Fungi” refers to PD agar, see materials and methods). (XLSX 30 kb)

Additional file 10: Figure S1. Maldi-Tof dendrogram of the obtained isolates, their isolation source and enrichment condition. (TIFF 1200 kb)

Additional file 11: Table S9. Details on the isolates. (XLSX 20 kb)

Additional file 12: Figure S2. Riboprint of all *Bacillus mycoides* isolates. (TIFF 207 kb)

Additional file 13: Figure S3. Boxplots of the results from quantitative PCR, targeting bacterial 16S rRNA genes (Y-axis: Gene copies per m2). Vertical line indicates the minimum and maximum of three replicates. The grey line reflects the median of three replicates, whereas the box indicates the 1st and third quartile. Each sampling campaign is indicated by a different colour. (TIFF 2627 kb)

Additional file 14: Figure S4. PCA plot depicting the relationships of cleanroom and changing room microbial communities (a) and communities before and after the spacecraft hardware was brought in (b) based on predicted function information. (TIFF 8400 kb)

Additional file 15: Figure S5. Network analysis of samples taken in September 2013. (TIFF 9424 kb)

Additional file 16: Figure S6. Network analysis of samples taken in December 2014. (TIFF 9538 kb)
