# Supplementary material for: Microbial biodiversity assessment of the European Space Agency’s ExoMars 2016 mission
Source: Microbiome. 2017 Oct 25;5:143. doi: 10.1186/s40168-017-0358-3 (PMC5657055; doi:10.1186/s40168-017-0358-3)
Supplement: Supplementary file 8 — Sequence data analysis pipelines. (DOCX 49 kb) [file 40168_2017_358_MOESM8_ESM.docx]

**16S rRNA Microbiota Data Analysis Against SILVA123 DB mothur in Galaxy**

| **Step 1: Assemble.paired**  Compressed directory with forward and reversed fastq/fastq.gz sequences  align - Select a pairwise alignment method  gotoh (default)  Trim with an oligos file?  no  match - Pairwise alignment reward for a match  1  mismatch - Pairwise alignment penalty for a mismatch  -1  gapopen - Pairwise alignment penalty for opening a gap  -2  gapextend - Pairwise alignment penalty for extending a gap  -1  Type of compressed directory  tar.gz |
| --- |
| **Step 2: Make.fastq**  fasta - Fasta Sequence file  Output dataset 'fasta' from step 1  qfile - Sequence Quality file  Output dataset 'qual' from step 1  choose what format your sequences are  False |
| **Step 3: Summary.seqs**  fasta - Dataset  Output dataset 'fasta' from step 1  name - Names  count - a count_table |
| **Step 4: Screen.seqs**  fasta - Fasta to screen  Output dataset 'fasta' from step 1  start - Remove sequences that start after position (ignored when negative)  -1  end - Remove sequences that end before position (ignored when negative)  -1  minlength - Remove sequences shorter than (ignored when negative)  200  maxlength - Remove sequences longer than (ignored when negative)  -1  maxambig - Remove sequences with ambiguous bases greater than (ignored when negative)  0  maxhomop - Remove sequences with homopolymers greater than (ignored when negative)  -1  criteria - Percent of sequences that an optimize value must match to be retained(ignored when negative)  -1  optimize - Optimize selected paramenters  Nothing selected.  qfile - Sequence Quality file to screen  Output dataset 'qual' from step 1  name - Sequence Names to screen  group - Groups to screen  Output dataset 'group' from step 1  alignreport - Align Report to screen  summary - allows you to enter the summary file created by summary.seqs to save processing time when screening with parameters in the summary file  contigsreport - file is created by the make.contigs command. If you provide the contigs report file you can screen your sequences using the following parameters: minoverlap, ostart, oend and mismatches  taxonomy - Taxonomy to screen  count - a count_table |
| **Step 5: FastQC**  Short read data from your current history  Output dataset 'fastq' from step 2  Contaminant list  Submodule and Limit specifing file |
| **Step 6: Make.fastq**  fasta - Fasta Sequence file  Output dataset 'out_file' from step 4  qfile - Sequence Quality file  Output dataset 'output_qfile' from step 4  choose what format your sequences are  False |
| **Step 7: Summary.seqs**  fasta - Dataset  Output dataset 'out_file' from step 4  name - Names  count - a count_table |
| **Step 8: Unique.seqs**  fasta - Sequences to filter  Output dataset 'out_file' from step 4  names - Sequences Names  count - a count_table |
| **Step 9: FastQC**  Short read data from your current history  Output dataset 'fastq' from step 6  Contaminant list  Submodule and Limit specifing file |
| **Step 10: Count.seqs**  name - Sequences Name reference  Output dataset 'output_names' from step 8  Use a Group file to include counts for groups  True  group - Group file for the tree  Output dataset 'output_groups' from step 4  groups - Groups to display  None  large - Datasets are large and may not fit in RAM  False |
| **Step 11: Align.seqs**  fasta - Candiate Sequences  Output dataset 'out_fasta' from step 8  Select Reference Template from  Cached Reference  reference - Select an alignment database  /home/galaxy/shed_tools/toolshed.g2.bx.psu.edu/repos/jjohnson/mothur_toolsuite/040410b8167e/mothur_toolsuite/mothur/tool-data/db/silva/silva.nr_v123.align  Select a search method  kmer (default)  ksize - kmer length between 5 and 12  8  align - Select a pairwise alignment method  needleman (default)  Alignment scoring values  use defaults  flip - Try to align against the reverse complement  No |
| **Step 12: Summary.seqs**  fasta - Dataset  Output dataset 'out_fasta' from step 8  name - Names  count - a count_table  Output dataset 'count_table' from step 10 |
| **Step 13: Summary.seqs**  fasta - Dataset  Output dataset 'out_file' from step 11  name - Names  count - a count_table  Output dataset 'count_table' from step 10 |
| **Step 14: Screen.seqs**  fasta - Fasta to screen  Output dataset 'out_file' from step 11  start - Remove sequences that start after position (ignored when negative)  13842  end - Remove sequences that end before position (ignored when negative)  23444  minlength - Remove sequences shorter than (ignored when negative)  -1  maxlength - Remove sequences longer than (ignored when negative)  -1  maxambig - Remove sequences with ambiguous bases greater than (ignored when negative)  -1  maxhomop - Remove sequences with homopolymers greater than (ignored when negative)  8  criteria - Percent of sequences that an optimize value must match to be retained(ignored when negative)  -1  optimize - Optimize selected paramenters  Nothing selected.  qfile - Sequence Quality file to screen  name - Sequence Names to screen  group - Groups to screen  alignreport - Align Report to screen  summary - allows you to enter the summary file created by summary.seqs to save processing time when screening with parameters in the summary file  contigsreport - file is created by the make.contigs command. If you provide the contigs report file you can screen your sequences using the following parameters: minoverlap, ostart, oend and mismatches  taxonomy - Taxonomy to screen  count - a count_table  Output dataset 'count_table' from step 10 |
| **Step 15: Filter.seqs**  fasta - Alignment Fasta  Output dataset 'out_file' from step 14  Additional Alignment Files  vertical - Vertical column  True  trump - Trump character  .  soft - percentage required to retain column. (0-100)  -1  hard - Hard Column Filter |
| **Step 16: Unique.seqs**  fasta - Sequences to filter  Output dataset 'out_fasta' from step 15  names - Sequences Names  count - a count_table  Output dataset 'output_count' from step 14 |
| **Step 17: Pre.cluster**  fasta - Sequence Fasta  Output dataset 'out_fasta' from step 16  name - Sequences Name reference or count_table  Output dataset 'output_count' from step 16  group (only needed in combination with names file) - Sequences Name reference  diffs - Number of mismatched bases to allow between sequences in a group (default 1)  3  allows you to specify whether to cluster from largest abundance to smallest or vice versa. Default =T, which is largest to smallest  False |
| **Step 18: Chimera.uchime**  fasta - Candiate Aligned Sequences  Output dataset 'fasta_out' from step 17  Select Reference Template from  Self count  abskew - Abundance skew (default 1.9)  1.9  count - a count_table  Output dataset 'output_count' from step 17  dereplicate - remove chimeric sequences from all groups, default=f  False  minh - mininum score to report chimera. Default 0.3  0.3  mindiv - minimum divergence ratio, default 0.5  0.5  xn - weight of a no vote. Default 8.0  8.0  dn - pseudo-count prior on number of no votes. Default 1.4  1.4  xa - eight of an abstain vote. Default 1.0  1.0  chunks - number of chunks. Default 4.  4  minchunk - minimum length of a chunk. Default 64.  64  idsmoothwindow - the length of id smoothing window. Default 32  32  maxp - maximum number of candidate parents to consider. Default 2  2  minlen - minimum unaligned sequence length. Default 10  0  maxlen - maximum unaligned sequence length. Defaults 10000  0  skipgaps - columns containing gaps do not count as diffs. Default=T  True  skipgaps2 - column is immediately adjacent to a column containing a gap, it is not counted as a diff. Default=T  True  chimealns - Produce a file containing multiple alignments of query sequences to parents in human readable format.  False  ucl - Use local-X alignments.  False |
| **Step 19: Remove.seqs**  accnos - Accession Names  Output dataset 'out_accnos' from step 18  fasta - Fasta Sequences  Output dataset 'fasta_out' from step 17  qfile - Fasta Quality  name - Sequences Name reference  group - Sequences Groups  alignreport - Align Report  list - OTU List  taxonomy - Taxonomy  dups - Apply to duplicates  True  count - a count_table  Output dataset 'output_count' from step 17  fastq |
| **Step 20: Classify.seqs**  fasta - Candiate Sequences  Output dataset 'fasta_out' from step 19  Select Reference Template from  Cached Reference  reference - Select an alignment database  /home/galaxy/shed_tools/toolshed.g2.bx.psu.edu/repos/jjohnson/mothur_toolsuite/040410b8167e/mothur_toolsuite/mothur/tool-data/db/trainset/trainset14_032015.pds/trainset14_032015.pds.fasta  Select Taxonomy from  Cached Reference  taxonomy - Taxonomy reference  /home/galaxy/shed_tools/toolshed.g2.bx.psu.edu/repos/jjohnson/mothur_toolsuite/040410b8167e/mothur_toolsuite/mothur/tool-data/db/trainset/trainset14_032015.pds/trainset14_032015.pds.tax  method - Select a classification method  Bayesian (default)  ksize - kmer length between 5 and 12  8  iters - iterations to do when calculating the bootstrap confidence score  100  cutoff - Confindence percentage cutoff between 1 and 100  80  probs - Show probabilities  True  count file  Output dataset 'output_count' from step 19  relabund - allows you to indicate that you want the summary files to be relative abundances rather than raw abundances. default=f  False |
| **Step 21: Summary.seqs**  fasta - Dataset  Output dataset 'fasta_out' from step 19  name - Names  count - a count_table  Output dataset 'output_count' from step 19 |
| **Step 22: Remove.lineage**  choose which file is used  taxonomy  taxonomy - Taxonomy  Output dataset 'taxonomy_out' from step 20  taxon - Select Taxons for filtering  Chloroplast-Mitochondria-unknown-Eukaryota  fasta - Fasta Sequences  Output dataset 'fasta_out' from step 19  group - Groups  alignreport - Align Report  list - OTU List  name - Sequences Name reference  dups - Apply to duplicate names  True  count - a count_table  Output dataset 'output_count' from step 19 |
| **Step 23: Cluster.split**  Split by  Classification using fasta  fasta - Sequences  Output dataset 'fasta_out' from step 22  name - Sequences Name reference  taxonomy - Taxonomy (from Classify.seqs)  Output dataset 'taxonomy_out' from step 22  taxlevel - taxonomy level for split (default=3)  4  classic - Use cluster.classic  False  count - a count_table  Output dataset 'output_count' from step 22  method - Select a Clustering Method  Average neighbor  cutoff - Distance Cutoff threshold - ignored if not > 0  0.15  hard - Use hard cutoff instead of rounding  True  precision - Precision for rounding distance values  .01  large - distance matrix is too large to fit in RAM  False  The cluster parameter allows you to indicate whether you want to run the clustering or just split the distance matrix, default=T.  False |
| **Step 24: Summary.seqs**  fasta - Dataset  Output dataset 'fasta_out' from step 22  name - Names  count - a count_table  Output dataset 'output_count' from step 22 |
| **Step 25: Classify.otu**  list - OTU List  Output dataset 'otulist' from step 23  name - taxonomy sequence names  count - used to represent the number of duplicate sequences for a given representative sequence  Output dataset 'output_count' from step 22  Select Taxonomy from  History  taxonomy - Taxonomy Reference  Output dataset 'taxonomy_out' from step 22  Select Reference Taxonomy used in Classify.seqs from  Selection is Optional  label - OTU Labels  None  cutoff - Confindence percentage cutoff between 1 and 100  80  probs - Show probabilities  True  basis - Summary file gives numbers of  OTU  group - Groups for summary file  persample - allows you to find a consensus taxonomy for each group. default=f  False |
| **Step 26: Make.shared**  list - OTU List  Output dataset 'otulist' from step 23  group - or count file  Output dataset 'output_count' from step 22  label - Select OTU Labels to include  None  groups - Groups to include  None  Create a new history dataset for each group rabund  False |
| **Step 27: Make.biom**  shared - OTU Shared file  Output dataset 'shared' from step 26  contaxonomy - consensus taxonomy  Output dataset 'contaxonomy' from step 25  metadata  use picrust program  False  matrixtype - sparse or dense  sparse  groups - Groups to include  None  label - Select OTU Labels to include  None |
| **Step 28: Make.biom**  shared - OTU Shared file  Output dataset 'shared' from step 26  contaxonomy - consensus taxonomy  Output dataset 'contaxonomy' from step 25  metadata  use picrust program  False  matrixtype - sparse or dense  sparse  groups - Groups to include  None  label - Select OTU Labels to include |

**##Network analyses##**

#BIOM filee was splitted into Dezember and September biom files with

filter samples from otu table

-i/--all.biom -m/--mapping.txt –keep_Dez.txt -o/-- biomDez.biom

# and also September

filter samples from otu table

-i/--all.biom -m/--mapping.txt –keep_Sep.txt -o/-- biomSep.biom

#OTU networks were generated separately with

Make out network

-i/-- biomDez.biom -m/--mapping_Dez.txt

-i/-- biomSep.biom -m/--mapping_Sep.txt

#use output for Cytoscape

#->Real edge table AND Real node table were used for Cytoscape

**Sanger sequence data pipeline mothur**

**summary.seqs**(fasta=File.fasta)

**screen.seqs**(fasta=File.fasta, group=File.groups, minlength=1000, maxlength=1700, maxhomop=8)

**summary.seqs**(fasta=File.good.fasta)

**unique.seqs**(fasta=File.good.fasta)

**count.seqs**(name=File.good.names, group=File.good.groups)

**align.seqs**(fasta=File.good.unique.fasta, reference=v123.txt, flip=t)

**summary.seqs**(fasta=File.good.unique.align)

**screen.seqs**(fasta=File.good.unique.align, name=File.good.names, group=File.good.groups, minlength=1000)

**summary.seqs**(fasta=File.good.unique.good.align)

**filter.seqs**(fasta=File.good.unique.good.align, vertical=T, trump=.)

**unique.seqs**(fasta=File.good.unique.good.filter.fasta, name=File.good.good.names, group=File.good.good.groups)

**chimera.uchime**(fasta=File.good.unique.good.filter.fasta, name=File.good.good.names, group=File.good.good.groups, dereplicate=t)

**summary.seqs**(fasta=File.good.unique.good.filter.fasta)

**classify.seqs**(fasta=File.good.unique.good.filter.fasta, name=File.good.good.names, group=File.good.good.groups, reference=trainset14_032015.pds.fasta, taxonomy=trainset14_032015.pds.tax, cutoff=80)

**remove.lineage**(fasta=File.good.unique.good.filter.fasta, name=File.good.good.names, group=File.good.good.groups, taxonomy=File.good.unique.good.filter.rdp.wang.taxonomy, taxon=unknown)

**count.seqs**(name=File.good.good.pick.names, group=File.good.good.pick.groups)

**cluster.split**(fasta=File.good.unique.good.filter.pick.fasta, count=File.good.good.pick.count_table, taxonomy=File.good.unique.good.filter.rdp.wang.pick.taxonomy, splitmethod=classify, taxlevel=4, cutoff=0.15)

**make.shared**(list=File.good.unique.good.filter.pick.an.unique_list.list, count=File.good.good.pick.count_table, label=0.03)

**classify.otu**(list=File.good.unique.good.filter.pick.an.unique_list.list, count=File.good.good.pick.count_table, taxonomy=File.good.unique.good.filter.rdp.wang.pick.taxonomy, label=0.03)

**make.biom**(shared=File.good.unique.good.filter.pick.an.unique_list.shared, constaxonomy=File.good.unique.good.filter.pick.an.unique_list.0.03.cons.taxonomy)

**16S rRNA Microbiota Data Analysis Against GreenGenes 13_8 DB Qiime in Galaxy**

| **Step 1: Input dataset**  input |
| --- |
| **Step 2: Input dataset**  input |
| **Step 3: Input dataset**  input |
| **Step 4: Count Seqs**  -i/--input_dir: Input fasta/fastq file or compressed fastq or fasta files. Please, compress all files together as .tar.gz. Don't use any directory!  Output dataset 'output' from step 1  File type: fasta, fastq or fastq.gz. [default:fastq]  fastq  --suppress_errors: Suppress warnings about missing files [default: False]  False |
| **Step 5: Multiple Join Paired Ends**  -i/--input_dir: Input directory of directories, or directory of paired fastq files.  Output dataset 'output' from step 1  -p/--parameter_fp: path to the parameter file, which specifies changes to the default behavior of join_paired_ends.py. See http://www.qiime.org/documentation/file_formats.html#qiime-parameters [default: join_paired_ends.py defaults will be used]  Output dataset 'output' from step 2  --read1_indicator: Substring to search for to indicate read 1 [default: _R1_]  Empty.  --read2_indicator: Substring to search for to indicate read 2 [default: _R2_]  Empty.  -b/--match_barcodes: Enable searching for matching barcodes [default: False]  False  --barcode_indicator: Substring to search for to indicate barcode reads [default: _I1_]  Empty.  --leading_text: Leading text to add to each join_paired_ends.py command [default: no leading text added]  Empty.  --trailing_text: Trailing text to add to each join_paired_ends.py command [default: no trailing text added]  Empty.  --include_input_dir_path: Include the input directory name in the output directory path. Useful in cases where the file names are repeated in input folders [default: False]  False  --remove_filepath_in_name: Disable inclusion of the input filename in the output directory names. Must use --include_input_dir_path if this option is enabled [default: False]  False  -w/--print_only: Print the commands but don't call them -- useful for debugging [default: False]  False  File type: fastq.gz or fastq. [default:fastq]  fastq |
| **Step 6: Count Seqs**  -i/--input_dir: Input fasta/fastq file or compressed fastq or fasta files. Please, compress all files together as .tar.gz. Don't use any directory!  Output dataset 'output_dir' from step 5  File type: fasta, fastq or fastq.gz. [default:fastq]  fastq  --suppress_errors: Suppress warnings about missing files [default: False]  False |
| **Step 7: Multiple Split Libraries Fastq**  -i/--input_dir: Input directory of directories or fastq files.  Output dataset 'output_dir' from step 5  -m/--demultiplexing_method: Method for demultiplexing. Can either be "sampleid_by_file" or "mapping_barcode_files". With the sampleid_by_file option, each fastq file (and/or directory name) will be used to generate the --sample_ids value passed to split_libraries_fastq.py. The mapping_barcode_files option will search for barcodes and mapping files that match the input read files [default: sampleid_by_file]  Selection is Optional  -p/--parameter_fp: path to the parameter file, which specifies changes to the default behavior of split_libraries_fastq.py. See http://www.qiime.org/documentation/file_formats.html#qiime-parameters [default: split_libraries_fastq.py defaults will be used]  Output dataset 'output' from step 2  --read_indicator: Substring to search for to indicate read files [default: _R1_]  Empty.  --barcode_indicator: Substring to search for to indicate barcode files [default: _I1_]  Empty.  --mapping_indicator: Substring to search for to indicate mapping files [default: _mapping_]  Empty.  --mapping_extensions: Comma-separated list of file extensions used to identify mapping files. Only applies when --demultiplexing_method is "mapping_barcode_files" [default: txt,tsv]  Empty.  --sampleid_indicator: Text in fastq filename before this value will be used as output sample ids [default: _]  Empty.  --include_input_dir_path: Include the input directory name in the output sample id name. Useful in cases where the file names are repeated in input folders [default: False]  True  --remove_filepath_in_name: Disable inclusion of the input filename in the output sample id names. Must use --include_input_dir_path if this option is enabled [default: False]  True  --leading_text: Leading text to add to each split_libraries_fastq.py command [default: no leading text added]  Empty.  --trailing_text: Trailing text to add to each split_libraries_fastq.py command [default: no trailing text added]  Empty.  -w/--print_only: Print the commands but don't call them -- useful for debugging [default: False]  False |
| **Step 8: Count Seqs**  -i/--input_dir: Input fasta/fastq file or compressed fastq or fasta files. Please, compress all files together as .tar.gz. Don't use any directory!  Output dataset 'output_dir' from step 7  File type: fasta, fastq or fastq.gz. [default:fastq]  fasta fna  --suppress_errors: Suppress warnings about missing files [default: False]  False |
| **Step 9: Cutadapt**  Fastq file to trim  Output dataset 'output_dir' from step 7  -m/--mapping_fp: the mapping filepath [optional]. If this is selected, primers will be taken from mapping file, otherwise please provide correct primers.  Output dataset 'output' from step 3  **3' Adapters**  **5' or 3' (Anywhere) Adapters**  **5' (Front) Adapters**  Maximum error rate  0.1  Match times  1  Minimum overlap length  3  Match Read Wildcards  False  Do Not Match Adapter Wildcards  False  Output filtering options  Default (no filtering)  Additional output options  Default  Additional modifications to reads  No Read Modifications |
| **Step 10: Cutadapt**  Fastq file to trim  Output dataset 'output' from step 9  -m/--mapping_fp: the mapping filepath [optional]. If this is selected, primers will be taken from mapping file, otherwise please provide correct primers.  Output dataset 'output' from step 3  **3' Adapters**  **5' or 3' (Anywhere) Adapters**  **5' (Front) Adapters**  Maximum error rate  0.1  Match times  1  Minimum overlap length  3  Match Read Wildcards  False  Do Not Match Adapter Wildcards  False  Output filtering options  Set Filters  Discard Trimmed Reads  False  Minimum length  1  Maximum length  1  Additional output options  Default  Additional modifications to reads  No Read Modifications |
| **Step 11: Count Seqs**  -i/--input_dir: Input fasta/fastq file or compressed fastq or fasta files. Please, compress all files together as .tar.gz. Don't use any directory!  Output dataset 'output' from step 10  File type: fasta, fastq or fastq.gz. [default:fastq]  fasta fna  --suppress_errors: Suppress warnings about missing files [default: False]  False |
| **Step 12: Identify Chimeric Seqs**  -i/--input_fasta_fp: path to the input fasta file  Output dataset 'output' from step 10  Select if you want to use custom or offered reference sequence  Nothing selected.  -t/--id_to_taxonomy_fp: Path to tab-delimited file mapping sequences to assigned taxonomy if selected 'custom'. Each assigned taxonomy is provided as a semicolon-separated list. For assignment with rdp, each assigned taxonomy must be exactly 6 levels deep. [default: None]  -t/--id_to_taxonomy_fp: Path to tab-delimited file mapping sequences to assigned taxonomy. Each assigned taxonomy is provided as a semicolon-separated list. For assignment with rdp, each assigned taxonomy must be exactly 6 levels deep. [default: gg_13_8_otus/taxonomy/97_otu_taxonomy.txt]  GreenGenes_13_8_taxonomy  -r/--refseqs_fp: Path to reference sequences to search against if selected 'custom' [default: None]  -r/--reference_fp: the reference sequences [default: GreenGenes_13_8_97_otus]  GreenGenes_13_8_97_otus  -a/--aligned_reference_seqs_fp: Path to (Py)Nast aligned reference sequences. REQUIRED when method ChimeraSlayer [default: None]  -b/--blast_db: Database to blast against. Must provide either --blast_db or --reference_seqs_fp when method is blast_fragments [default: None]  -m/--chimera_detection_method: Chimera detection method. Choices: blast_fragments or ChimeraSlayer or usearch61. [default:ChimeraSlayer]  usearch61  -n/--num_fragments: Number of fragments to split sequences into (i.e., number of expected breakpoints + 1) [default: 3]  Not available.  -d/--taxonomy_depth: Number of taxonomic divisions to consider when comparing taxonomy assignments [default: 4]  Not available.  -e/--max_e_value: Max e-value to assign taxonomy [default: 1e-30]  Not available.  -R/--min_div_ratio: min divergence ratio (passed to ChimeraSlayer). If set to None uses ChimeraSlayer default value. [default: None]  Not available.  -k/--keep_intermediates: Keep intermediate files, useful for debugging [default: False]  False  --suppress_usearch61_intermediates: Use to suppress retention of usearch intermediate files/logs.[default: False]  False  --suppress_usearch61_ref: Use to suppress reference based chimera detection with usearch61 [default: False]  False  --suppress_usearch61_denovo: Use to suppress de novo based chimera detection with usearch61 [default: False]  False  --split_by_sampleid: Enable to split sequences by initial SampleID, requires that fasta be in demultiplexed format, e.g., >Sample.1_0, >Sample.2_1, >Sample.1_2, with the initial string before first underscore matching SampleIDs. If not in this format, could cause unexpected errors. [default: False]  False  --non_chimeras_retention: usearch61 only - selects subsets of sequences detected as non-chimeras to retain after de novo and reference based chimera detection. Options are intersection or union. union will retain sequences that are flagged as non-chimeric from either filter, while intersection will retain only those sequences that are flagged as non-chimeras from both detection methods. [default: union]  Empty.  --usearch61_minh: Minimum score (h). Increasing this value tends to reduce the number of false positives and decrease sensitivity.[default: 0.28]  Not available.  --usearch61_xn: Weight of 'no' vote. Increasing this value tends to the number of false positives (and also sensitivity). Must be > 1.[default: 8.0]  Not available.  --usearch61_dn: Pseudo-count prior for 'no' votes. (n). Increasing this value tends to the number of false positives (and also sensitivity). Must be > 0.[default: 1.4]  Not available.  --usearch61_mindiffs: Minimum number of diffs in a segment. Increasing this value tends to reduce the number of false positives while reducing sensitivity to very low-divergence chimeras. Must be > 0.[default: 3]  Not available.  --usearch61_mindiv: Minimum divergence, i.e. 100% - identity between the query and closest reference database sequence. Expressed as a percentage, so the default is 0.8, which allows chimeras that are up to 99.2% similar to a reference sequence. This value is chosen to improve sensitivity to very low-divergence chimeras. Must be > 0.[default: 0.8]  Not available.  --usearch61_abundance_skew: Abundance skew setting for de novo chimera detection with usearch61. Must be > 0. [default: 2.0]  Not available.  --percent_id_usearch61: Percent identity threshold for clustering with usearch61, expressed as a fraction between 0 and 1. [default: 0.97]  Not available.  --minlen: Minimum length of sequence allowed for usearch61 [default: 64]  Not available.  --word_length: word length value for usearch61. [default: 8]  Not available.  --max_accepts: max_accepts value to usearch61. [default: 1]  Not available.  --max_rejects: max_rejects value for usearch61. [default: 8]  Not available.  --threads: Specify number of threads per core to be used for usearch61 commands that utilize multithreading. By default, will calculate the number of cores to utilize so a single thread will be used per CPU. Specify a fractional number, e.g. 1.0 for 1 thread per core, or 0.5 for a single thread on a two core CPU. Only applies to usearch61. [default: one_per_cpu]  10 |
| **Step 13: Filter Fasta**  -f/--input_fasta_fp: path to the input fasta file  Output dataset 'output' from step 10  -m/--otu_map: An OTU map where sequences ids are those which should be retained.  -s/--seq_id_fp: A list of sequence identifiers (or tab-delimited lines with a seq identifier in the first field) which should be retained.  Output dataset 'output_fp' from step 12  -b/--biom_fp: A biom file where otu identifiers should be retained.  -a/--subject_fasta_fp: A fasta file where the seq ids should be retained.  -p/--seq_id_prefix: Keep seqs where seq_id starts with this prefix.  Empty.  --sample_id_fp: Keep seqs where seq_id starts with a sample id listed in this file. Must be newline delimited and may not contain a header.  -n/--negate: Discard passed seq ids rather than keep passed seq ids. [default: False]  True  --mapping_fp: Mapping file path (for use with --valid_states). [default: None]  --valid_states: Description of sample ids to retain (for use with --mapping_fp). [default: None]  Empty.  If you are using compressed directory, please select BIOM table you want to analyze:  Nothing selected.  If you are using compressed directory, please final_otu_map you want to analyze:  Nothing selected. |
| **Step 14: Count Seqs**  -i/--input_dir: Input fasta/fastq file or compressed fastq or fasta files. Please, compress all files together as .tar.gz. Don't use any directory!  Output dataset 'output_fasta_fp' from step 13  File type: fasta, fastq or fastq.gz. [default:fastq]  fasta fna  --suppress_errors: Suppress warnings about missing files [default: False]  False |
| **Step 15: Pick Open Reference OTUs**  **input_fps**  **input_fps 1**  -i/--input_fps: the input sequences filepath or comma-separated list of filepaths  Output dataset 'output_fasta_fp' from step 13  -m/--otu_picking_method: The OTU picking method to use for reference and de novo steps. Passing usearch61, for example, means that usearch61 will be used for the de novo steps and usearch61_ref will be used for reference steps. [default: uclust]  Selection is Optional  Select if you want to use custom or offered reference sequence  Nothing selected.  -t/--id_to_taxonomy_fp: Path to tab-delimited file mapping sequences to assigned taxonomy if selected 'custom'. Each assigned taxonomy is provided as a semicolon-separated list. For assignment with rdp, each assigned taxonomy must be exactly 6 levels deep. [default: None]  -r/--refseqs_fp: Path to reference sequences to search against if selected 'custom' [default: None]  -r/--reference_seqs_fp: Path to reference sequences (if offered was selected). For assignment with blast, these are used to generate a blast database. For assignment with rdp, they are used as training sequences for the classifier. [default: gg_13_8_otus/rep_set/97_otus.fasta]  GreenGenes_13_8_97  -p/--parameter_fp: path to the parameter file, which specifies changes to the default behavior. See http://www.qiime.org/documentation/file_formats.html#qiime-parameters . [if omitted, default values will be used]  Output dataset 'output' from step 2  --prefilter_refseqs_fp: the reference sequences to use for the prefilter, if different from the reference sequecnces to use for the OTU picking [default: same as passed for --reference_fp]  -n/--new_ref_set_id: Unique identifier for OTUs that get created in this ref set (this is useful to support combining of reference sets) [default:New]  Empty.  -a/--parallel: Run in parallel where available [default: False]  True  -O/--jobs_to_start: Number of jobs to start. NOTE: you must also pass -a to run in parallel, this defines the number of jobs to be started if and only if -a is passed [default: 1]  10  -s/--percent_subsample: Percent of failure sequences to include in the subsample to cluster de novo, expressed as a fraction between 0 and 1 (larger numbers should give more comprehensive results but will be slower) [default:0.001]  Not available.  --prefilter_percent_id: Sequences are pre-clustered at this percent id (expressed as a fraction between 0 and 1) against the reference and any reads which fail to hit are discarded (a quality filter); pass 0.0 to disable [default:0.0]  Not available.  --step1_otu_map_fp: reference OTU picking OTU map, to avoid rebuilding if one has already been built. This must be an OTU map generated by this workflow, not (for example) pick_closed_reference_otus.py.  --step1_failures_fasta_fp: reference OTU picking failures fasta filepath, to avoid rebuilding if one has already been built. This must be a failures file generated by this workflow, not (for example) pick_closed_reference_otus.py.  --minimum_failure_threshold: The minimum number of sequences that must fail to hit the reference for subsampling to be performed. If fewer than this number of sequences fail to hit the reference, the de novo clustering step will run serially rather than invoking the subsampled open reference approach to improve performance. [default: 100000]  Not available.  --suppress_step4: suppress the final de novo OTU picking step (may be necessary for extremely large data sets) [default: False]  False  --min_otu_size: the minimum otu size (in number of sequences) to retain the otu [default: 2]  Not available.  --suppress_taxonomy_assignment: skip the taxonomy assignment step, resulting in an OTU table without taxonomy [default: False]  False  --suppress_align_and_tree: skip the sequence alignment and tree-building steps [default: False]  False |
| **Step 16: Convert from BIOM to TXT**  -i/Path to input biom file.  Output dataset 'output_dir' from step 15  Convert biom format to classic format, including the taxonomy observation metadata as the last column of the classic format table.  True  Convert biom format to classic format, including the taxonomy observation metadata as the last column of the classic format table, but renaming that column as ConsensusLineage.  True  Select BIOM table you want to convert:  otu_table_mc2_w_tax_no_pynast_failures |
| **Step 17: Create summary of BIOM table**  -i/Path to input biom file.  Output dataset 'output_dir' from step 15  Select BIOM table you want to convert:  otu_table_mc2_w_tax_no_pynast_failures |
| **Step 18: List of files from directory**  Select dataset to extract:  Output dataset 'output_dir' from step 15 |
| **Step 19: Core Diversity Analyses**  -i/--input_biom_fp: the input biom file [REQUIRED]  Output dataset 'output_dir' from step 15  -m/--mapping_fp: the mapping filepath [REQUIRED]  Output dataset 'output' from step 3  -e/--sampling_depth: Sequencing depth to use for even sub-sampling and maximum rarefaction depth. If you live this field empty, it will automatically take 'minimal sample count' value from biom summarize-table.  0  -p/--parameter_fp: path to the parameter file, which specifies changes to the default behavior. For more information, see www.qiime.org/documentation/qiime_parameters_files.html [if omitted, default values will be used]  Output dataset 'output' from step 2  -a/--parallel: Run in parallel where available. Specify number of jobs to start with -O or in the parameters file. [default: False]  True  --nonphylogenetic_diversity: Apply non-phylogenetic alpha (chao1 and observed_otus) and beta (bray_curtis) diversity calculations. This is useful if, for example, you are working with non-amplicon BIOM tables, or if a reliable tree is not available (e.g., if you're working with ITS amplicons) [default: False]  False  --suppress_taxa_summary: Suppress generation of taxa summary plots. [default: False]  False  --suppress_beta_diversity: Suppress beta diversity analyses. [default: False]  False  --suppress_alpha_diversity: Suppress alpha diversity analyses. [default: False]  False  --suppress_group_significance: Suppress OTU/category significance analysis. [default: False]  False  -t/--tree_fp: Path to the tree file if one should be used. Required unless --nonphylogenetic_diversity is passed. [default: no tree will be used]  Output dataset 'output_dir' from step 15  -c/--categories: The metadata category or categories to compare (i.e., column headers in the mapping file) for categorical analyses. These should be passed as a comma-separated list. [default: None; do not perform categorical analyses]  Empty.  -w/--print_only: Print the commands but don't call them -- useful for debugging or recovering from failed runs. [default: False]  False  --recover_from_failure: Don't fail if output directory exists, but attempt to recover from the failed run. [default: False]  False  -O/--jobs_to_start: Number of jobs to start. NOTE: you must also pass -a to run in parallel, this defines the number of jobs to be started if and only if -a is passed [default: 1]  10  If you are using compressed directory, please select BIOM table you want to analyze:  otu_table_mc2_w_tax_no_pynast_failures |
| **Step 20: Extract**  Select dataset to extract:  Output dataset 'output_dir' from step 15  Select files you want to extract:  Output dataset 'output_fp' from step 18  Select files  otu_table_mc2_w_tax_no_pynast_failures.biom |
| **Step 21: Show Core Diversity Results**  Short results from privious step  Output dataset 'output_dir' from step 19 |

**PICRUSt on Open OTUs**

| **Step 1: Input dataset**  input |  |
| --- | --- |
| **Step 2: Filter OTUs from OTU Table**  -i/--input_fp: the input otu table filepath in biom format  Output dataset 'output' from step 1  --negate_ids_to_exclude: keep OTUs in otu_ids_to_exclude_fp rather than discard them [default:False]  True  -n/--min_count: the minimum total observation count of an otu for that otu to be retained [default: 0]  Not available.  --min_count_fraction: fraction of the total observation (sequence) count to apply as the minimum total observation count of an otu for that otu to be retained. this is a fraction, not percent, so if you want to filter to 1%, you specify 0.01. [default: 0]  Not available.  -x/--max_count: the maximum total observation count of an otu for that otu to be retained [default: infinity]  Not available.  -s/--min_samples: the minimum number of samples an OTU must be observed in for that otu to be retained [default: 0]  Not available.  -y/--max_samples: the maximum number of samples an OTU must be observed in for that otu to be retained [default: infinity]  Not available.  -e/-otu_ids_to_exclude_fp: file containing list of OTU ids to exclude: can be a text file with one id per line, a text file where id is the first value in a tab-separated line, or can be a fasta file (extension must be .fna or .fasta) [default: None]  If you didn't provide own list of OTUs to exclude you must select one of the databases. [default: None]  Nothing selected.  If you are using compressed directory, please select BIOM table you want to analyze:  otu_table_mc2_w_tax_no_pynast_failures |  |
| **Step 3: Convert from HDF5 to JSON**  -i/Path to input biom file.  Output dataset 'output_fp' from step 2  Select BIOM table you want to convert:  otu_table_mc2_w_tax_no_pynast_failures |  |
| **Step 4: Normalize by Copy Number**  -i/--input_otu_fp: the input otu table filepath in biom format  Output dataset 'output_fp' from step 3  -g/--gg_version: Version of GreenGenes that was used for OTU picking. Valid choices are: 13_5, 18may2012 [default: 13_5]  Selection is Optional  -c/--input_count_fp: Precalculated input marker gene copy number predictions on per otu basis in biom format (can be gzipped).Note: using this option overrides --gg_version. [default: None]  --metadata_identifer: identifier for copy number entry as observation metadata [default: CopyNumber]  Empty.  -f/--input_format_classic: input otu table (--input_otu_fp) is in classic Qiime format [default: False]  False  --load_precalc_file_in_biom: Instead of loading the precalculated file in tab-delimited format (with otu ids as row ids and traits as columns) load the data in biom format (with otu as SampleIds and traits as ObservationIds) [default: False]  False |  |
| **Step 5: Predict Metagenomes**  -i/--input_otu_table: the input otu table in biom format  Output dataset 'output_otu_fp' from step 4  -t/--type_of_prediction: Type of functional predictions. Valid choices are: ko, cog, rfam [default: ko]  Selection is Optional  -g/--gg_version: Version of GreenGenes that was used for OTU picking. Valid choices are: 13_5, 18may2012 [default: 13_5]  Selection is Optional  -c/--input_count_table: Precalculated function predictions on per otu basis in biom format (can be gzipped). Note: using this option overrides --type_of_prediction and --gg_version. [default: None]  --suppress_subset_loading: Normally, only counts for OTUs present in the sample are loaded. If this flag is passed, the full biom table is loaded. This makes no difference for the analysis, but may result in faster load times (at the cost of more memory usage)  False  --load_precalc_file_in_biom: Instead of loading the precalculated file in tab-delimited format (with otu ids as row ids and traits as columns) load the data in biom format (with otu as SampleIds and traits as ObservationIds) [default: False]  False  --input_variance_table: Precalculated table of variances corresponding to the precalculated table of function predictions. As with the count table, these are on a per otu basis and in BIOM format (can be gzipped). Note: using this option overrides --type_of_prediction and --gg_version. [default: None]  --with_confidence: Calculate 95% confidence intervals for metagenome predictions. By default, this uses the confidence intervals for the precalculated table of genes for greengenes OTUs. If you pass a custom count table with -c and select this option, you must also specify a corresponding table of confidence intervals for the gene content prediction using --input_variance_table. (these are generated by running predict_traits.py with the --with_confidence option). If this flag is set, three addtional output files will be generated, named the same as the metagenome prediction output, but with .variance .upper_CI or .lower_CI appended immediately before the file extension[default: False]  False  -f/--format_tab_delimited: output the predicted metagenome table in tab-delimited format [default: False]  False |  |
| **Step 6: Categorize by Function**  -i/--input_fp: the predicted metagenome table  Output dataset 'output_metagenome_table' from step 5  -c/--metadata_category: the metadata category that describes the hierarchy: KEGG_Pathways or taxonomy. [default: KEGG_Pathways]  KEGG_Pathways  -l/--level: the level in the hierarchy to collapse to. A value of 0 is not allowed, a value of 1 is the highest level, and any higher value nears the leaves of the hierarchy. For instance, if the hierarchy contains 4 levels, specifying 3 would collapse at one level above being fully specified.  3  --ignore: Ignore the comma separated list of names. For instance, specifying --ignore_unknown=unknown,unclassified will ignore those labels while collapsing. The default is to not ignore anything. [default: None]  Empty.  -f/--format_tab_delimited: output the predicted metagenome table in tab-delimited format [default: False]  False |  |
