# Supplementary figures and images for: Microbial biodiversity assessment of the European Space Agency’s ExoMars 2016 mission
Source: Microbiome. 2017 Oct 25;5:143. doi: 10.1186/s40168-017-0358-3 (PMC5657055; doi:10.1186/s40168-017-0358-3)

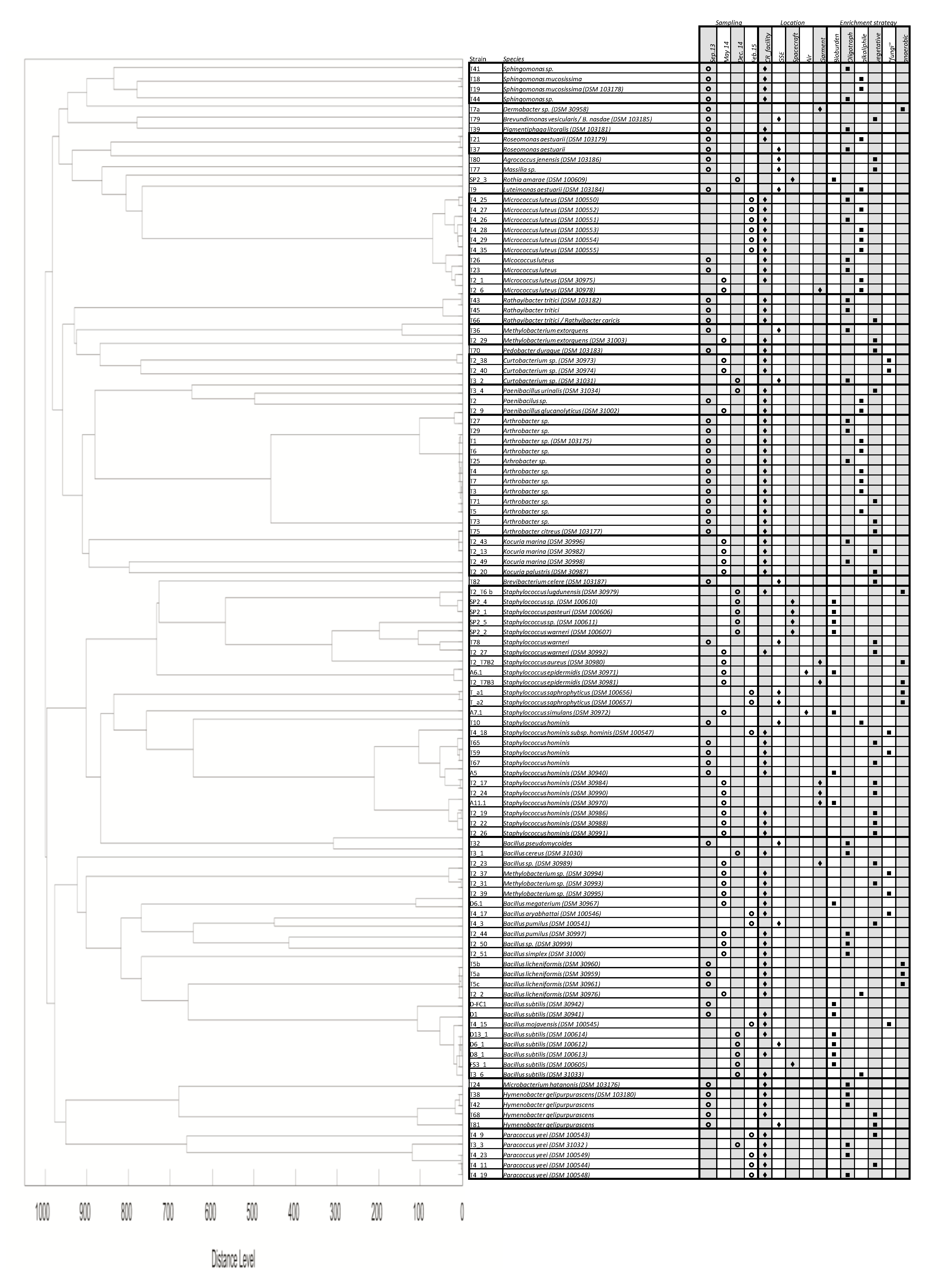

Supplement: Supplementary file 10 — Maldi-Tof dendrogram of the obtained isolates, their isolation source and enrichment condition. (TIFF 1200 kb) [file 40168_2017_358_MOESM10_ESM.tif]

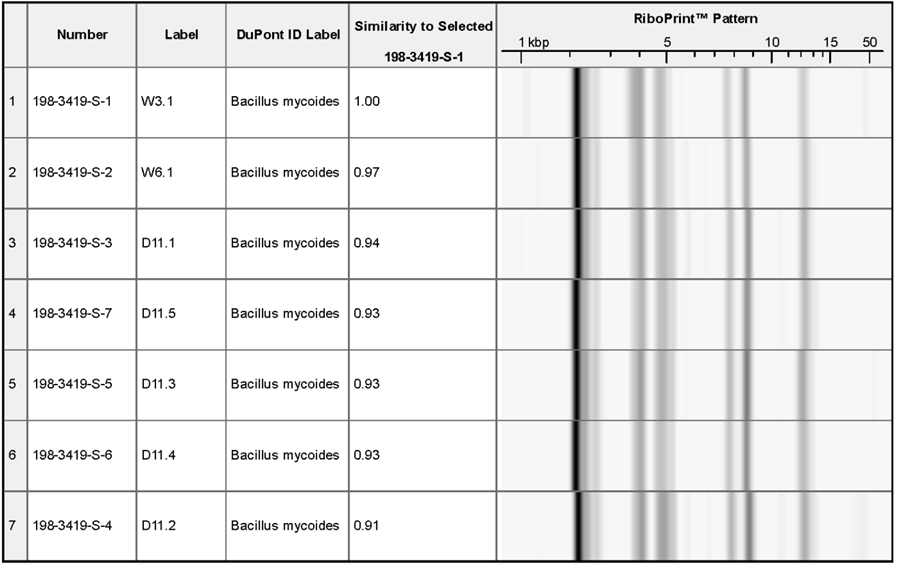

Supplement: Supplementary file 12 — Riboprint of all Bacillus mycoides isolates. (TIFF 207 kb) [file 40168_2017_358_MOESM12_ESM.tif]

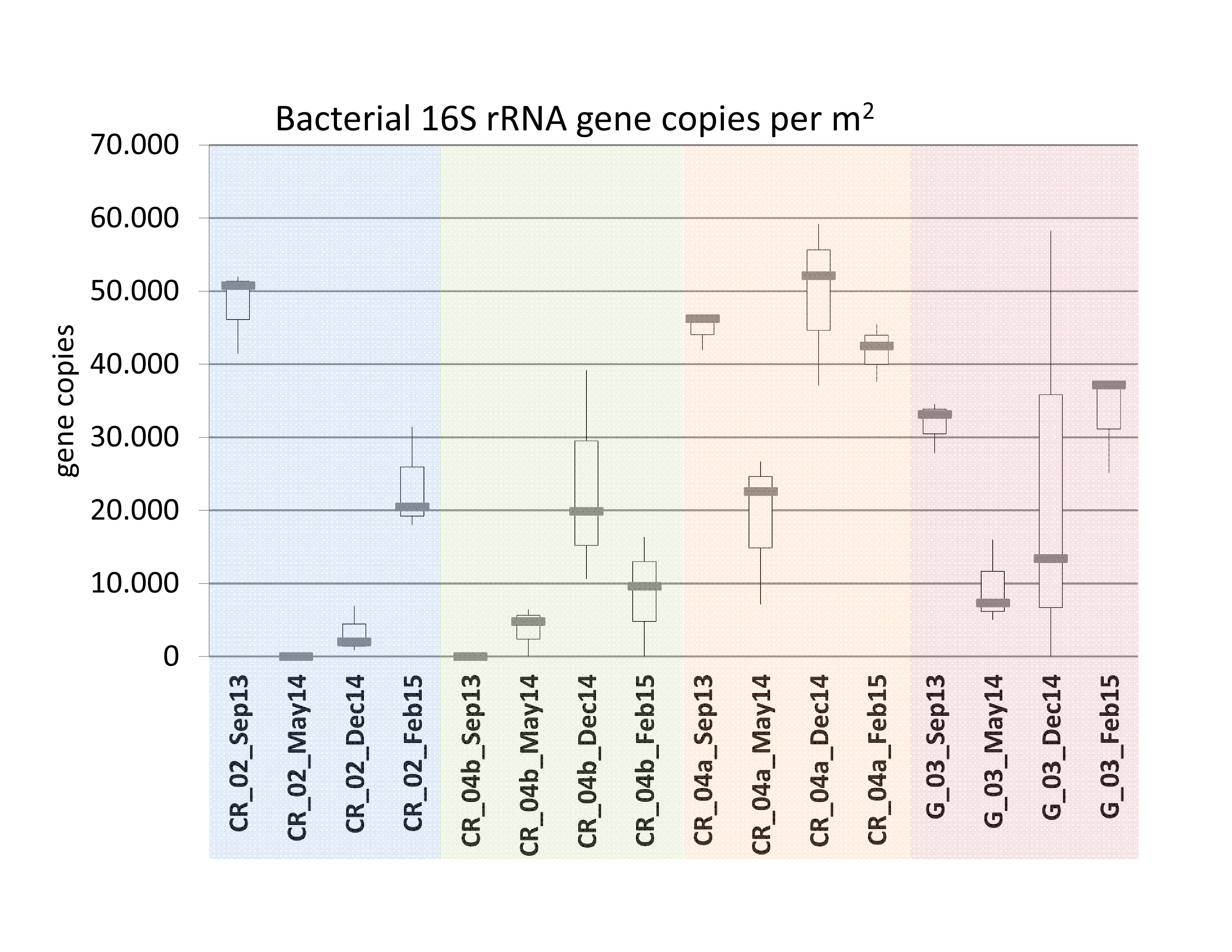

Supplement: Supplementary file 13 — Boxplots of the results from quantitative PCR, targeting bacterial 16S rRNA genes (Y-axis: Gene copies per m2). Vertical line indicates the minimum and maximum of three replicates. The grey line reflects the median of three replicates, whereas the box indicates the 1st and third quartile. Each sampling campaign is indicated by a different colour. (TIFF 2627 kb) [file 40168_2017_358_MOESM13_ESM.tif]

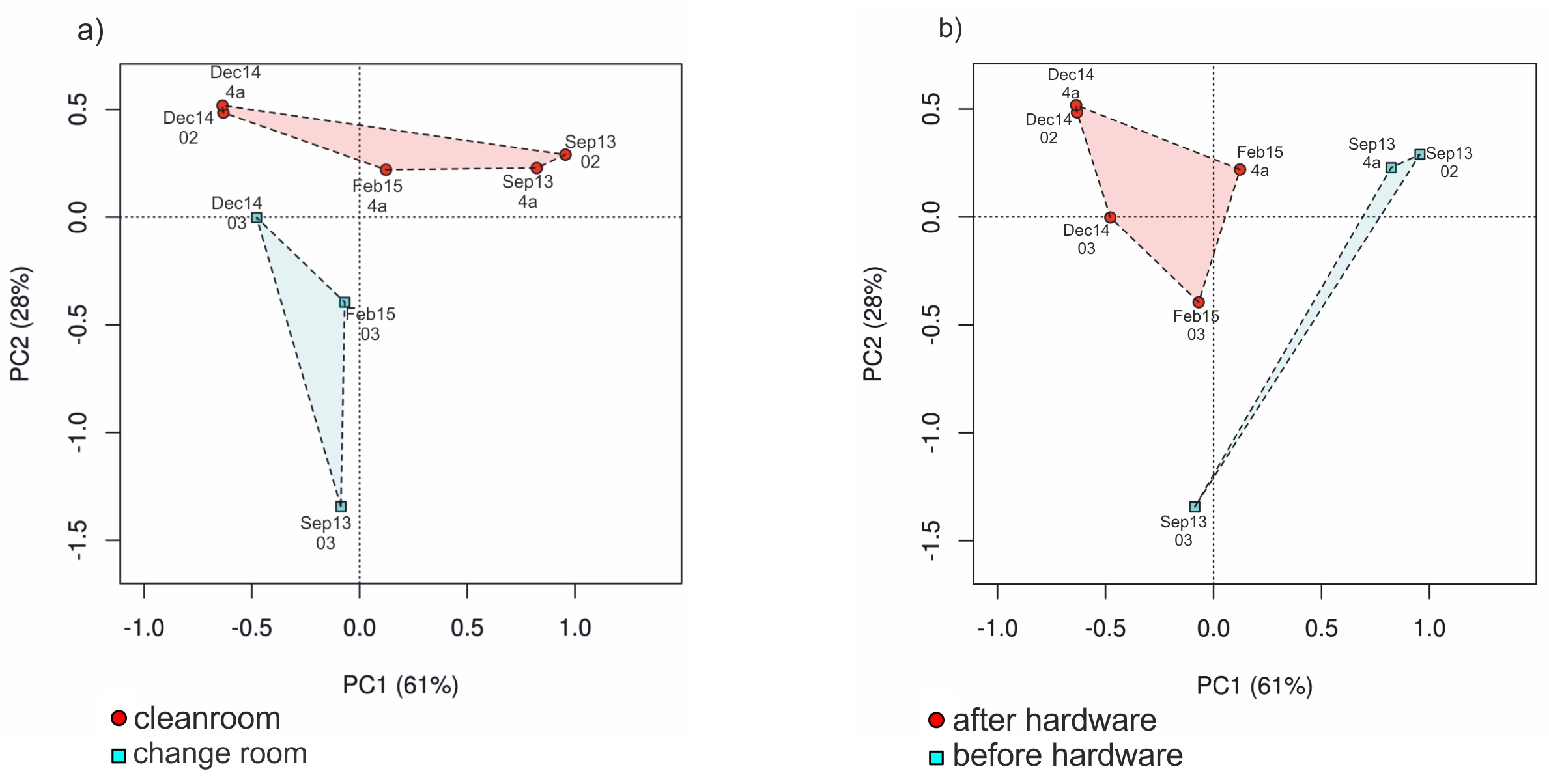

Supplement: Supplementary file 14 — PCA plot depicting the relationships of cleanroom and changing room microbial communities (a) and communities before and after the spacecraft hardware was brought in (b) based on predicted function information. (TIFF 8400 kb) [file 40168_2017_358_MOESM14_ESM.tif]
